# Transcriptome signature analysis repurposes trifluoperazine for the treatment of fragile X syndrome in mouse model

**DOI:** 10.1038/s42003-020-0833-4

**Published:** 2020-03-16

**Authors:** Qi Ding, Ferzin Sethna, Xue-Ting Wu, Zhuang Miao, Ping Chen, Yueqi Zhang, Hua Xiao, Wei Feng, Yue Feng, Xuan Li, Hongbing Wang

**Affiliations:** 1 Department of Physiology, Michigan State University, East Lansing, USA; 2 Genetics Program, Michigan State University, East Lansing, USA; 3 Key Laboratory of Synthetic Biology, Institute of Plant Physiology and Ecology, Shanghai Institutes for Biological Sciences, Chinese Academy of Sciences, 200032 Shanghai, China; 4 Department of Pharmacology and Chemical Biology, Emory University School of Medicine, Atlanta, GA 30322 Georgia; 5 Neuroscience Program, Michigan State University, East Lansing, MI 48824 USA

**Keywords:** Neuroscience, Computational biology and bioinformatics

## Abstract

Fragile X syndrome (FXS) is a prevailing genetic disorder of intellectual disability and autism. There is no efficacious medication for FXS. Through in silico screening with a public database, computational analysis of transcriptome profile in FXS mouse neurons predicts therapeutic value of an FDA-approved drug trifluoperazine. Systemic administration of low-dose trifluoperazine at 0.05 mg/kg attenuates multiple FXS- and autism-related behavioral symptoms. Moreover, computational analysis of transcriptome alteration caused by trifluoperazine suggests a new mechanism of action against PI3K (Phosphatidylinositol-4,5-bisphosphate 3-kinase) activity. Consistently, trifluoperazine suppresses PI3K activity and its down-stream targets Akt (protein kinase B) and S6K1 (S6 kinase 1) in neurons. Further, trifluoperazine normalizes the aberrantly elevated activity of Akt and S6K1 and enhanced protein synthesis in FXS mouse. Together, our data demonstrate a promising value of transcriptome-based computation in identification of therapeutic strategy and repurposing drugs for neurological disorders, and suggest trifluoperazine as a potential treatment for FXS.

## Abstract (web summary)

Qi Ding, Ferzin Sethna et al. perform a computational analysis of the transcriptome profile of *Fmr1*^−/−^ neurons and identify trifluoperazine as potential therapeutic agent against Fragile X Syndrome. Next, they show that low doses of trifluoperazine ameliorate some of the behavioral and molecular phenotypes present in *Fmr1*^−/−^ mice.

## Introduction

Fragile X syndrome (FXS), caused by mutations in the *FMR1* (fragile X mental retardation 1) gene, is the most common form of inherited intellectual disability and a leading cause of autism^1^. As the most prevailing form of mutation, expansion of CGG repeats in the 5′ un-translated region of the *FMR1* gene inhibits its transcription and thereby preventing the expression of its gene product FMRP (fragile X mental retardation protein). FXS patients exhibit numerous neurological abnormalities including intellectual disability, social impairment, perseveration, and hyperactivity^2^. Despite recent advances in the understanding of FMRP function, FXS pathophysiology, and identification of potential therapeutic targets^3^, development of practical therapy has had limited success. As there is no efficacious medication to treat FXS, identification of practical therapeutic intervention is of urgent demand.

Recent studies with transcriptome landscapes in distinct neuronal populations suggest that gene signature may represent a holistic molecular outcome and an indicator for cell type classification and function^4^. As a molecular phenotype and pathological endpoint, transcriptome alteration has been observed in patient samples from major psychiatric disorders^5,6^. Intriguingly, using transcriptome landscape as a holistic outcome may offer an unbiased approach to reveal disease mechanism as well as neuropathology and etiology that are unique or overlapping among psychiatric disorders^7^. Recent studies also suggest that analysis of disease-associated transcriptome profile may also predict potential therapy or aid treatment development. For example, antipsychotics-induced transcriptome changes are negatively correlated with those identified in schizophrenia samples. In contrast, psychotomimetic phencyclidine triggers transcriptome changes that overlap with the disease profile^7^. Regarding its potential application in drug discovery, comparison of psychiatric disease-associated transcriptomes with drug-induced transcript alterations in public database can computationally reveal the previously validated drug-disease pairs^8^. However, whether the transcriptome-based computational approach can help identify new therapeutic or repurposing drugs for new application is of great interest but remains to be examined^8^.

In this study, we identified significant transcriptome changes in *Fmr1* deficient neurons. We compared the transcriptome signature in *Fmr1* knockout (KO) neuron with those in the connectivity Map (CMAP) database^9^, which contains over 7000 reference gene expression profiles representing transcriptome changes affected by 1309 compounds/drugs. Computational analysis outcome predicts therapeutic value of an FDA-approved drug trifluoperazine. Using the FXS mouse model, we found significant effects of trifluoperazine on correcting FXS-associated and autism-associated symptoms. Further analysis with drug-induced transcriptome changes revealed a new pharmacologically inhibitory activity of trifluoperazine against PI3K (Phosphatidylinositol-4,5-bisphosphate 3-kinase). Administration of trifluoperazine normalizes the aberrantly elevated PI3K/Akt (protein kinase B)-S6K1 (S6 kinase 1) signaling in FXS. Our data support the value of computational transcriptome analysis in therapeutic development, and suggest trifluoperazine as a potential practical medication for FXS treatment.

## Results

### Screening of CMAP database with FXS transcriptome signature

**Fig. 1:**
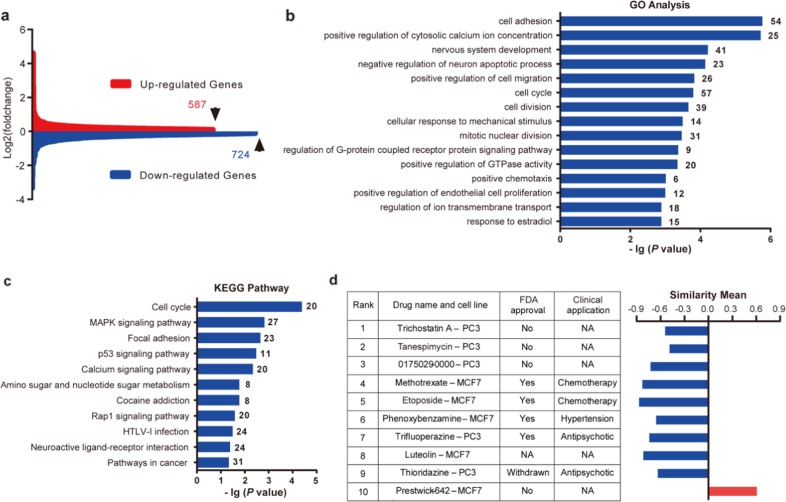
RNA-seq and connectivity Map (CMAP) analysis of differentially expressed genes (DEG) in Fmr1 knockout (KO) hippocampal neuron. **a** Gene expression differences between wild type (WT) and *Fmr1* KO samples. There are 587 upregulated and 724 downregulated genes in *Fmr1* KO hippocampal neuron. **b** Top 15 GO biology processes that are associated with DEG between WT and *Fmr1* KO samples. **c** KEGG pathways that are associated with DEG between WT and *Fmr1* KO samples. **d** Top-ranked 10 CMAP compounds/drugs that induce transcriptome alterations oppositional to (indicated by negative similarity mean) or overlapping with (indicated by positive similarity mean) that caused by *Fmr1* deficiency. Rank is determined by *P* value and enrichment score. Drug name and cell line indicate the name of compound used for treatment with specific cell lines in CMAP database. Full information of *P* value, enrichment score, and similarity mean is shown in Supplementary Data 5.

Transcriptome alteration represents an emerging molecular phenotype and pathological signature of chronic diseases including psychiatric disorders^5–8^. Here, we used next generation sequencing (NGS) to determine genome-wide changes of gene transcript in *Fmr1* KO hippocampal neurons. Compared to wild type (WT) neurons, 587 gene transcripts were upregulated and 724 were downregulated in *Fmr1* KO samples (Fig. 1a and Supplementary Data 1). Enrichment analysis identified significant changes in 138 GO processes (complete list in Supplementary Data 2; top 15 pathways in Fig. 1b and Supplementary Data 3) and 11 KEGG pathways (Fig. 1c and Supplementary Data 4). Some enrichment groups are related to neural development, calcium homeostasis and signaling, p53 signaling, Rap1 signaling, and MAPK signaling (Fig. 1b, c), the alterations of which are implicated in FXS^10–13^. As alterations of numerous transcripts occur in multiple pathways (Supplementary Data 3, 4), it is challenging to pinpoint which specific DEG (differentially expressed gene) plays a causal role in FXS pathology.

Recent computational analysis with transcriptomes imputed from GWAS data and CMAP drug-induced gene signature suggests an intriguing strategy to identify potential treatment for psychiatric disorders^8^. The strategy, which identifies therapeutic compounds inducing gene signature oppositional to disease signature (indicated by negative similarity mean), was supported by known medications used for psychiatric conditions. For example, predicted therapeutic compounds for schizophrenia are enriched for antipsychotics^8^. To examine the value of this unbiased approach, we compared transcriptome signature of *Fmr1* KO neurons with drug-induced signatures in CMAP^9^. We identified negative and positive correlations between the FXS-drug pair. Among the top ten hits ranked by *p* value and enrichment, nine compounds/drugs induce oppositional transcriptome changes (as indicated by negative similarity mean) (Fig. 1d and Supplementary Data 5), predicating potential therapeutic value. From the four FDA-approved drugs, we chose trifluoperazine, which can effectively cross the blood brain barrier (BBB)^14,15^ and has been used to treat central nervous system disorders, for experimental validation of therapeutic efficacy. The other three FDA-approved compounds have been used for cancer chemotherapy and hypertension treatment, respectively, and were not selected for the current study.

### Trifluoperazine corrects FXS-associated behaviors in mice

Previous studies demonstrate that efficacy doses of trifluoperazine used for schizophrenia and anxiety show extrapyramidal side effects in patients^16^, but low dose trifluoperazine is well tolerated in both human and animal models^17^. We examined the key FXS-associated behavioral symptoms following low dose i.p. injection (at 0.05 mg/kg).

**Fig. 2:**
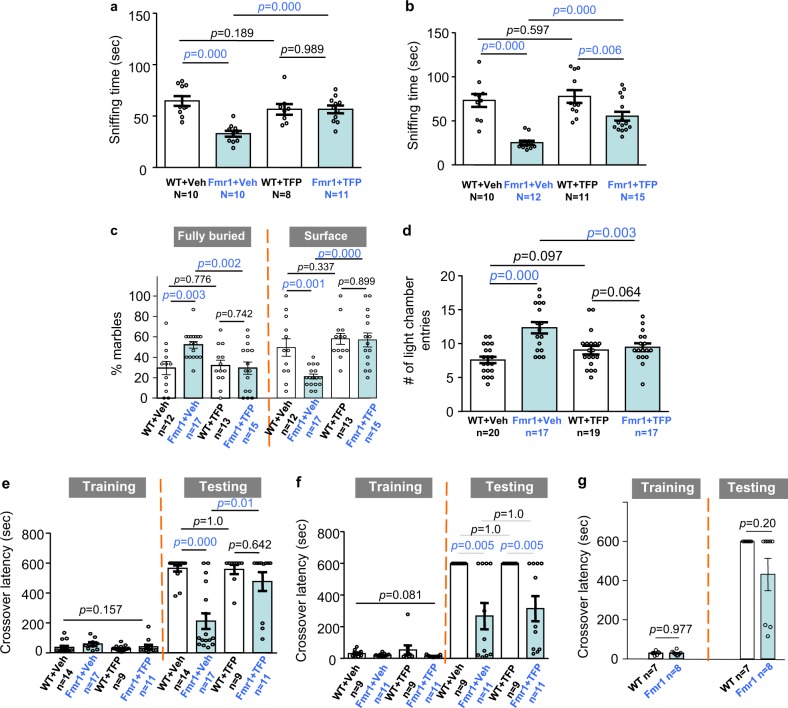
Trifluoperazine corrects FXS- and autism-associated behavioral symptoms in Fmr1 KO mice. Trifluoperazine (TFP, at 0.05 mg/kg) or vehicle (Veh) was administered into wild type (WT) and *Fmr1* KO mice (*Fmr1*) 1 h before testing (**a**–**d**). **a**, **b** TFP improves sociability in *Fmr1* KO and does not affect WT mice. **c** The percentage of fully buried marbles and marbles on the surface (as indicated) was determined for WT and *Fmr1* KO mice following TFP or vehicle administration. **d** TFP corrects the increased locomotive transition between the lit and dark chamber in *Fmr1* KO mice. The total number of entries to the lit chamber was scored. **e** Mice were injected with vehicle or trifluoperazine 60 min before passive avoidance training. Mice were tested 24 h after training, and crossover latency was scored. **f** Mice were injected with vehicle or trifluoperazine 60 min after passive avoidance training. Mice were tested 24 h after training, and crossover latency was scored. There is no difference in crossover latency among the four groups during training (genotype effect: *F*_1,36_ = 3.218, *p* = 0.081; drug effect: *F*_1,36_ = 0.309, *p* = 0.581; genotype × drug interaction: *F*_1,36_ = 1.256, *p* = 0.270). *Fmr1* KO mice showed impaired memory and not corrected by trifluoperazine. **g** Mice received three mild electric foot shocks during passive avoidance training. Mice were tested 24 h after training, and crossover latency was scored. **a**–**d** *p* values between the indicated two groups were determined by two-way ANOVA followed by Holm-Sidak test. **e**, **f** Two-way ANOVA and Fisher’s exact test was used to analyze training and testing data, respectively. **g** Student’s *t*-test and Fisher’s exact test was used to analyze training and testing data, respectively.

We first examined the effects of trifluoperazine on sociability, reduction of which prevalently occurs in FXS and autism. We used two different 3-chamber social interaction paradigms, both of which have been applied in previous studies and detected social impairment in *Fmr1* KO mice^18,19^. The first paradigm measures social interaction without presenting an un-animated object (Fig. 2a); the second paradigm involves both social (i.e., a stranger mouse) and un-animated objects (Fig. 2b). We confirmed that, in both paradigms, *Fmr1* KO mice showed less social interaction than WT mice (genotype effect: *F*_1,35_ = 14.851, *p* = 0.000 in Fig. 2a; genotype effect: *F*_1,44_ = 38.690, *p* = 0.000 in Fig. 2b). Trifluoperazine did not affect WT mice but significantly improved sociability in *Fmr1* KO mice (Fig. 2a; treatment effect: *F*_1,35_ = 3.475, *p* = 0.071; genotype × treatment interaction: *F*_1,35_ = 14.704, *p* = 0.001) (Fig. 2b; treatment effect: *F*_1,44_ = 9.364, *p* = 0.004; genotype × treatment interaction: *F*_1,44_ = 5.119, *p* = 0.029).

*Fmr1* KO mice show significant repetitive/stereotypic behavior, recapitulating perseverative symptoms in FXS and autism patients. We examined animal behavior in marble-burying test, which has been considered to measure repetitive behavior and barely involves novelty-induced anxiety^20^. We found that trifluoperazine had no effect on WT but normalized the excessive marble burying behavior in *Fmr1* KO mice (for fully buried marbles in Fig. 2c; genotype effect: *F*_1,53_ = 3.726, *p* = 0.059; treatment effect: *F*_1,53_ = 3.808, *p* = 0.056; genotype × treatment interaction: *F*_1,53_ = 5.758, *p* = 0.02; post-hoc Holm-Sidak test detected significant difference between vehicle-treated WT and *Fmr1* KO mice) (for marbles on the surface in Fig. 2c; genotype effect: *F*_1,53_ = 6.255, *p* = 0.016; treatment effect: *F*_1,53_ = 14.217, *p* = 0.000; genotype × treatment interaction: *F*_1,53_ = 5.383, *p* = 0.024; post-hoc Holm-Sidak test detected significant difference between vehicle-treated WT and *Fmr1* KO mice). The number of marbles partially buried by WT and *Fmr1* KO mice was similar (Supplementary Fig. 1; genotype effect: *F*_1,53_ = 2.01, *p* = 0.162). Due to overall increase in combined number of fully buried and surface marbles, the number of partially buried marbles was decreased in both WT and *Fmr1* KO mice injected with trifluoperazine (Supplementary Fig. 1; drug effect: *F*_1,53_ = 11.393, *p* = 0.001; genotype–drug interaction: *F*_1,53_ = 0.096, *p* = 0.758). We further examined *Fmr1* KO mice with light-dark test. The *Fmr1* KO mice showed more transition between the light and dark chambers than WT animals, indicating both hyperactivity and repetitive behavior (Fig. 2d; genotype effect: *F*_1,69_ = 16.69, *p* = 0.000). *Fmr1* KO and WT mice spent comparable time in the light and dark chamber (genotype effect: *F*_1,69_ = 0.428, *p* = 0.515; treatment effect: *F*_1,69_ = 0.046, *p* = 0.830; genotype × treatment interaction: genotype effect: *F*_1,69_ = 0.820, *p* = 0.368) (Supplementary Fig. 2). Trifluoperazine corrected the excessive transition in *Fmr1* KO mice but did not affect WT mice (Fig. 2d; treatment effect: *F*_1,69_ = 1.276, *p* = 0.263; genotype-treatment interaction: *F*_1,69_ = 11.73, *p* = 0.001). In other stereotypic behavior tests, *Fmr1* KO mice displayed normal self-grooming (Supplementary Fig. 3) but excessive digging behavior (Supplementary Fig. 4). Trifluoperazine did not show significant effect on correcting excessive digging (Supplementary Fig. 4).

Hyperactivity is observed in both human FXS patients and *Fmr1* KO mice^21,22^. In a novel open field arena, we confirmed that *Fmr1* KO mice have enhanced locomotor activity in the whole arena (Supplementary Fig. 5a, b; genotype effect: *F*_1,28_ = 24.226, *p* = 0.000) as well as in the center area (Supplementary Fig. 5c, d; genotype effect: *F*_1,28_ = 17.106, *p* = 0.000). We did not detect significant effect of trifluoperazine on locomotion in the whole arena (drug effect: *F*_1,28_ = 0.762, *p* = 0.39; genotype × drug interaction: *F*_1,28_ = 1.592, *p* = 0.217) or the center area (drug effect: *F*_1,28_ = 1.183, *p* = 0.286; genotype × drug interaction: *F*_1,28_ = 0.376, *p* = 0.545).

FXS is the leading cause of inherited intellectual disability. To determine certain aspect of cognitive deficits, we examined passive avoidance memory. Trifluoperazine or vehicle was administered before training, during which animals received an electric foot shock once entering the dark chamber. Twenty-four hours after training, animals were tested for memory formation, which is implicated by increased latency to cross over and enter the dark chamber. All groups of animals showed similar crossover latency during training (Fig. 2e, genotype effect: *F*_1,47_ = 2.069, *p* = 0.157; drug effect: *F*_1,47_ = 2.424, *p* = 0.126; genotype–drug interaction: *F*_1,47_ = 0.248, *p* = 0.569). During testing, vehicle-treated WT mice showed significantly longer crossover latency than vehicle-treated *Fmr1* KO mice (Fig. 2e, *p* = 0.000, Fisher’s exact test). This confirms the impaired passive avoidance memory in the FXS mice^21,22^. Although trifluoperazine did not have an effect on WT animals, crossover latency in trifluoperazine-treated *Fmr1* KO mice significantly increased to the WT level (Fig. 2e, *p* = 0.01, Fisher’s exact test). We expect that the improved memory in *Fmr1* KO mice is not due to trifluoperazine effect on locomotion. This is because the memory test was performed 24 h after trifluoperazine administration. When tested in a similar context (i.e., movement in the light-dark box test), the effect of trifluoperazine on locomotion is evident 1 h (Fig. 2d) but not 24 h after drug administration (Supplementary Fig. 6; genotype effect: *F*_1,22_ = 18.631, *p* = 0.000; drug effect: *F*_1,22_ = 0.325, *p* = 0.574; genotype × drug interaction: *F*_1,22_ = 0.166, *p* = 0.688 for the number of transition between the lit and dark chambers). Interestingly, when trifluoperazine was administered after training, it had no significant effect on correcting the defective passive avoidance memory in *Fmr1* KO mice (Fig. 2f). This suggests an intriguing possibility that lack of FMRP causes impaired learning but not memory consolidation. Supportively, when trained by a stronger learning paradigm, during which three consecutive electric foot shocks were delivered, *Fmr1* KO mice showed normal passive avoidance memory (Fig. 2g).

Hyper sensitivity and susceptibility to seizures are common in FXS patients. Here, we confirmed that WT mice do not show audiogenic seizure (AGS), which is a major behavioral phenotype in FXS mouse model^23^. While *Fmr1* KO mice receiving trifluoperazine displayed reduction in AGS, the drug effect is marginal but not significant (Supplementary Fig. 7; *p* = 0.113 for wild running and *p* = 0.273 for seizure).

### Effects of repeated trifluoperazine on behavioral symptoms

**Fig. 3:**
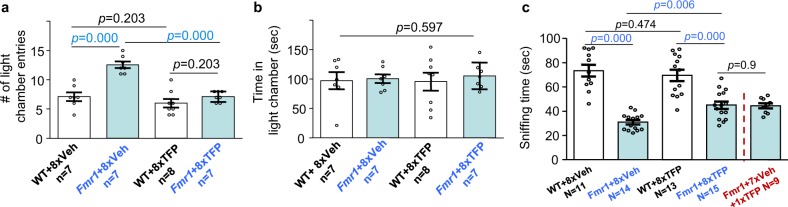
Repeated administration of trifluoperazine (TFP) does not cause desensitization to the drug. Wild type (WT) and *Fmr1* KO mice (Fmr1) were injected with TFP (0.05 mg/kg) or vehicle daily for 8 days. Mice were subjected to light-dark test (**a**, **b**) or social interaction test (determined by paradigm 1) (**c**) 1 h after the last injection. In a separate *Fmr1* KO cohort, mice were treated with seven daily injection of vehicle and 1 TFP injection on day 8. Then, social interaction was examined 1 h after TFP injection (**c**). For light-dark test, number of locomotive transition between the light and dark chamber (**a**) and time spent in the light chamber (**b**) are presented. For social interaction, total time spent in sniffing the novel stimulus mouse enclosure is presented (**c**). The *p* values were determined by two-way ANOVA followed by Holm-Sidak test. Student’s *t*-test was used to compare *Fmr1* KO mice receiving eight TFP injections and mice receiving seven vehicle and 1 TFP injection in **c**.

One potential problem related to treatment is drug desensitization following repeated administration. Trifluoperazine or vehicle was administered once per day for 8 days. We examined light-dark test and social interaction 1 hour after the last drug administration. While the vehicle-treated *Fmr1* KO mice showed more transitions between the two chambers, the trifluoperazine-treated group showed fewer transitions, which were comparable to the WT level (Fig. 3a; genotype effect: *F*_1,25_ = 27.303, *p* = 0.000; drug effect: *F*_1,25_ = 27.303, *p* = 0.000; genotype × drug interaction: *F*_1,25_ = 11.613, *p* = 0.002). Repeated trifluoperazine had no effect on the time spent in each chamber (Fig. 3b; genotype effect: *F*_1,25_ = 0.287, *p* = 0.597; drug effect: *F*_1,25_ = 0.019, *p* = 0.892; genotype × drug interaction: *F*_1,25_ = 0.079, *p* = 0.781).

In another independent behavioral paradigm, repeated trifluoperazine improved social interaction in *Fmr1* KO mice (Fig. 3c). However, the treated-*Fmr1* KO mice still showed less social interaction than WT mice (Fig. 3c; genotype effect: *F*_1,49_ = 83.685, *p* = 0.000; drug effect: *F*_1,49_ = 1.896, *p* = 0.175; genotype × drug interaction: *F*_1,49_ = 5.985, *p* = 0.018). This is likely due to that sociability in *Fmr1* KO mice is more sensitive to the repeated injection procedure (Supplementary Fig. 8). With another cohort of *Fmr1* KO mice, we found that 7 repeated vehicle injections followed by one trifluoperazine injection had similar effect to that of eight repeated trifluoperazine injections (Fig. 3c). These data indicate that *Fmr1* KO mice are not desensitized to trifluoperazine following repeated exposures to the drug.

### Trifluoperazine normalizes the elevated protein synthesis

**Fig. 4:**
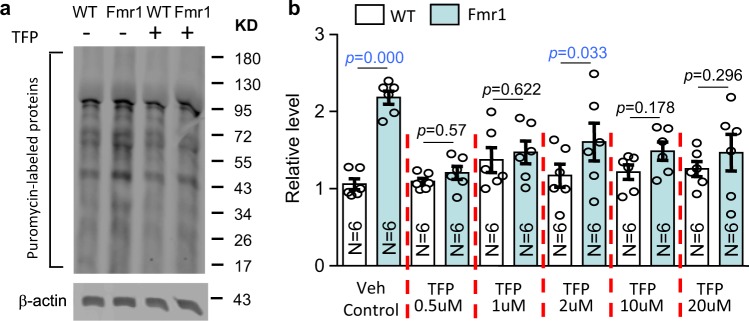
Trifluoperazine corrects enhanced basal translation in Fmr1 KO neurons. Basal protein synthesis was determined in wild type (WT) and *Fmr1* KO (Fmr1) mouse neurons by the SUnSET method. DIV 14 primary hippocampal neurons were pre-incubated with vehicle (Veh) or trifluoperazine (TFP) (at various concentrations as indicated) for 30 min before the addition of puromycin into the culture media. Equal amounts of protein were loaded on the gel for Western blot analysis with anti-puromycin antibody. **a** Representative image shows that the level of puromycin-labeled proteins is higher in *Fmr1* KO than WT neurons, and trifluoperazine dampens protein synthesis in *Fmr1* KO but not WT neurons. **b** Quantification shows that the enhanced protein synthesis in *Fmr1* KO but not WT neurons was suppressed by trifluoperazine. The relative level of newly synthesized puromycin-labeled protein in the vehicle-treated WT group was defined as 1, and all samples were normalized to this group. The *p* values were determined by two-way ANOVA followed by Holm-Sidak test.

Enhanced basal protein synthesis is thought to be the core cellular abnormality associated with FXS^1,3,24^. Here, we labeled newly synthesized proteins in neurons with puromycin using the SUnSET method^18,25^. We observed enhanced protein synthesis in *Fmr1* KO neurons compared to WT neurons (Fig. 4a, b; genotype effect: *F*_1,60_ = 21.249, *p* = 0.000). Treatment with trifluoperazine specifically reduced the level of puromycin-labeled proteins in *Fmr1* KO neurons to the WT level; it did not affect protein synthesis in WT neurons (Fig. 4; drug effect: *F*_1,60_ = 2.286, *p* = 0.057; genotype × drug interaction: *F*_5,60_ = 3.733, *p* = 0.005).

### CMAP predicts new activities of trifluoperazine

Aberrant intracellular signaling is considered as a prevailing disease mechanism that is causally related to both behavioral symptoms and elevated protein synthesis in FXS^13,18,26–28^. Regarding the mechanism of action (MOA), trifluoperazine is a phenothiazine derivative and has inhibition activity against dopamine receptors including both D1-like and D2-like receptors. Interestingly, a previous study showed hypo-dopaminergic function in FXS and that a dopamine receptor agonist rescued behavioral abnormality in *Fmr1* KO mice^29^. Thus, therapeutic effects of trifluoperazine are unlikely mediated through its inhibition action against dopamine receptors.

To identify potential new pharmacological activity of trifluoperazine, we screened CMAP for similarity compounds that induce transcriptome alterations overlapping with trifluoperazine-induced changes. Querying CMAP database using trifluoperazine signature in PC3 cell (Supplementary Data 6), which displays oppositional profile to that of *Fmr1* KO neurons (Fig. 1d), revealed compounds with positive and negative similarity scores (Supplementary Data 7). Similarity compounds were ranked in a descending order according to *p* value, enrichment and similarity mean (connectivity/similarity score). Among the top ten hits, four compounds are phenothiazine derivatives and used as typical antipsychotics (Table 1). The other two classes of compound over-represented among the top 10 hits are HDAC (histone deacetylase) inhibitors (i.e., vorinostat and trichostatin A) and PI3K signaling inhibitors (i.e., sirolimus and LY-294002). Further, another well-known PI3K inhibitor wortmannin also triggers transcriptome changes that significantly overlap with trifluoperazine-induced changes. Wortmannin-induced gene signatures in different cell lines are ranked at 35 (MCF7 cell, *p* = 0.00038, enrichment = 0.608, and similarity mean = 0.244) and 74 (PC3 cell, *p* = 0.00455, enrichment = 0.95, and similarity mean = 0.389), respectively (Supplementary Data 7). These results predict an intriguing possibility that trifluoperazine may process new pharmacological activity as HDAC and/or PI3K signaling inhibitor.

**Table 1:** Top 10 trifluoperazine similarity compounds.

Rank	Compound name and cell line	Similarity mean	*N*	Enrichment	*p* value	Application/MOA
1	Fluphenazine—PC3	0.558	3	0.997	0	Typical antipsychotics/derivatives of phenothiazine
2	Thioridazine—PC3	0.569	5	0.994	0	Typical antipsychotics/derivatives of phenothiazine
3	Vorinostat—MCF7	0.333	7	0.92	0	HDAC inhibitor
4	Sirolimus—PC3	0.344	8	0.871	0	PI3K/Akt/mTOR inhibitor
5	Prochlorperazine—MCF7	0.369	9	0.867	0	Typical antipsychotics/derivatives of phenothiazine
6	Trichostatin A—PC3	0.347	55	0.862	0	HDAC inhibitor
7	Trifluoperazine—MCF7	0.371	9	0.787	0	Typical antipsychotics/derivatives of phenothiazine
8	Geldanamycin—MCF7	0.294	10	0.753	0	HSP inhibitor
9	LY-294002—PC3	0.315	12	0.748	0	PI3K/Akt/mTOR inhibitor
10	Trichostatin A—MCF7	0.29	92	0.729	0	HDAC inhibitor

Signature probes of trifluoperazine in PC3 cell are used as query to search similarity compounds in CMAP database. Compound with positive and negative similarity mean triggers overlapping and oppositional transcriptome changes, respectively, comparing to the change caused by trifluoperazine in PC3 cell. Rank, *p* value, enrichment score, and similarity mean are described in Supplementary Data 5. Compound name and cell line indicate the name of compound used for treatment with specific cell lines in CMAP database. Number of arrays (*N*) is the number of analyzed arrays obtained from all instances of the corresponding compound. MOA: mechanism of action.

### Trifluoperazine inhibits the PI3K-Akt-S6K1 cascade

**Fig. 5:**
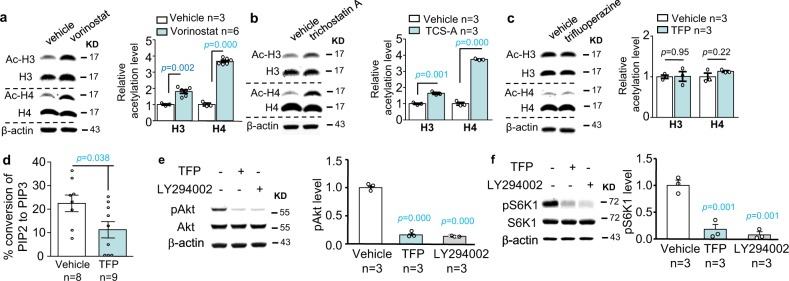
Trifluoperazine suppresses the PI3K-Akt-S6K1 signaling cascade but does not affect histone acetylation in hippocampal neurons. **a**–**c** Hippocampal neurons cultured from WT mice were treated with vorinostat (20 μM) (**a**), trichostatin A (20 μM) (**b**), and trifluoperazine (20 μM) (**c**) for 1 h, and then harvested for Western blot analysis. Level of the acetylated histone H3 and H4 (Ac-H3 and Ac-H4) in vehicle-treated and drug-treated sample was normalized to total H3 and H4, respectively. **d** PI3 kinase activity in lysates from primary WT hippocampal neurons treated with 20 µM trifluoperazine (TFP) or vehicle was determined by ELISA, which measures the concentration of PIP3 converted from PIP2. **e**, **f** Primary WT hippocampal neurons were treated with trifluoperazine (TFP) (20 µM) or LY-294002 (20 µM) for 1 h. Samples harvested immediately after drug or vehicle treatment were analyzed by Western blot for the level of pAkt (normalized to total Akt) (**e**) and pS6K1 (normalized to total S6K1) (**f**). The relative protein level in the vehicle-treated control group was defined as 1 (**a**–**c**, **e**, **f**). The *p* values (**a**–**d**) were determined by student’s *t*-test. The *p* value between the treated and control samples (**e**, **f**) was determined by one-way ANOVA and post hoc Tukey’s test (**e**, **f**).

We first examined the effect of trifluoperazine on histone acetylation. We confirmed that treatment with vorinostat (Fig. 5a) or trichostatin A (Fig. 5b) increased acetylation of histone H3 and H4. We did not observe any significant effects of trifluoperazine on these readouts of HDAC activity (Fig. 5c).

We next examined the effect of trifluoperazine on PI3K, elevated activity of which has been found in FXS samples^27,30^. Enzymatic assay demonstrates that trifluoperazine significantly suppresses PI3K activity in hippocampal neurons (Fig. 5d). We further examined the major PI3K downstream target Akt, and found that, similar to LY-294002, trifluoperazine also dampened Akt activity, as indicated by reduction of phosphorylation (Fig. 5e, treatment effect: *F*_2,6_ = 1200.316, *p* = 0.000, one-way ANOVA). We next examined the activity of S6K1, which has been implicated as PI3K/Akt downstream target and involved in regulating protein translation machinery and hyper-active in *Fmr1* KO mice and FXS patients^18,26,31^. We found that both LY-294002 and trifluoperazine suppressed S6K1 phosphorylation at Thr389 (a target site of PI3K^32,33^) (Fig. 5f, treatment effect: *F*_2,6_ = 37.441, *p* = 0.000, one-way ANOVA).

One complication is that, as implicated in mesangial cells, long-term trifluoperazine treatment has been shown to cause cell death, which may indirectly affect the activity of survival-supporting molecules such as Akt^34^. In our experimental set-up, 1-h trifluoperazine treatment did not affect neuronal viability (Supplementary Fig. 9; genotype effect: *F*_1,39_ = 3.547, *p* = 0.067; trifluoperazine effect: *F*_2,39_ = 1.580, *p* = 0.219; genotype × trifluoperazine interaction: *F*_2,39_ = 0.024, *p* = 0.976). These data demonstrate that trifluoperazine processes a new and previously un-described MOA against the PI3K-Akt-S6K1 signaling cascade in neurons.

**Fig. 6:**
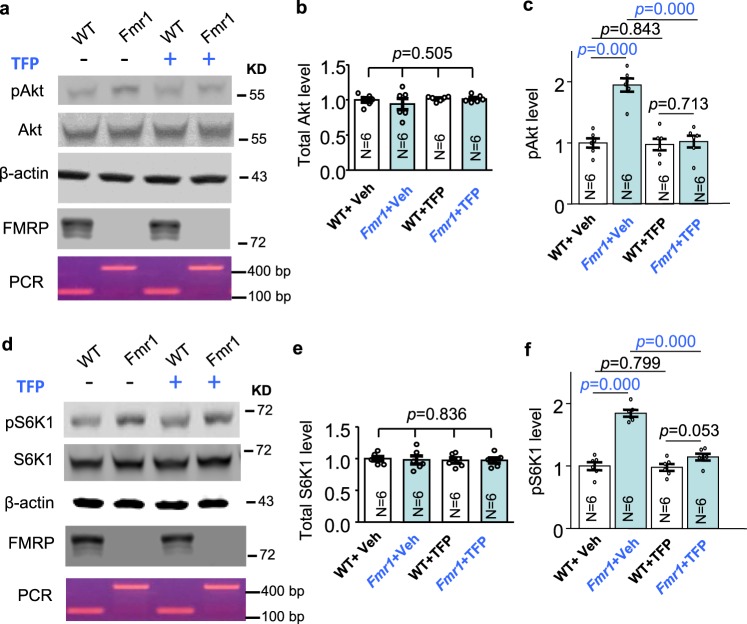
Trifluoperazine (TFP) corrects the aberrantly elevated Akt and S6K1 activity in Fmr1 KO mice. Wild type (WT) and *Fmr1* KO (Fmr1) mice were injected with vehicle or trifluoperazine (0.05 mg/kg) 1 h before hippocampus was harvested. The levels of total Akt (**a**, **b**) and phosphorylated Akt (pAkt) (**a**, **c**) as well as total S6K1 (**d**, **e**) and phosphorylated S6K1 (pS6K1 at Thr389) (**d**, **f**) were determined by Western blot. Protein loading was determined by the level of β-actin (**a**, **d**). The expression of FMRP was detected in WT but not *Fmr1* KO samples by Western blot (**a**, **d**). The genomic differences in WT and *Fmr1* KO samples were further confirmed by PCR-base genotyping; the PCR product is 130 bp for WT and 420 bp for *Fmr1* KO samples (the bottom panel **a**, **d**). Representative images are shown in **a**, **d**. For quantification, the level of total Akt (**b**) and total S6K1 (**e**) was normalized to the level of β-actin. The level of pAkt (**c**) and pS6K1 (**f**) was normalized to the level of total Akt and total S6K1, respectively. The relative level of Akt (or pAkt) and S6K1 (or pS6K1) in the vehicle-treated wild type (WT) group was defined as 1, and all samples were normalized to this group. The *p* values were determined by two-way ANOVA (**b**, **e**) or two-way ANOVA followed by Holm-Sidak test (**c**, **f**).

**Fig. 7:**
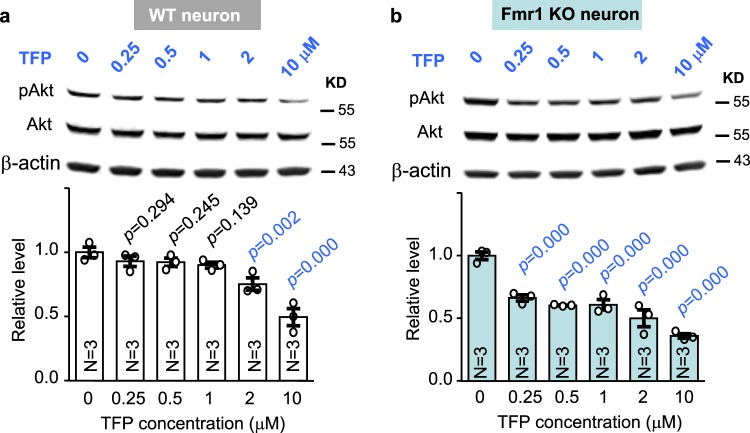
Fmr1 KO neurons are more sensitive to trifluoperazine treatment. DIV 14 wild type (WT) (**a**) and *Fmr1* KO neurons (**b**) were treated with trifluoperazine (TFP) at various concentrations (as indicated). Following the 1 h treatment, levels of pAkt, total Akt, and β-actin were determined by Western blot. The level of pAkt is normalized to the level of total Akt. The relative level of pAkt in the vehicle-treated control group is defined as 1, and all samples are normalized to this group. The *p* value between the indicated group and the vehicle-treated control group was determined by one-way ANOVA (*F*_5,12_ = 17.777, *p* = 0.000 for **a**; *F*_5,12_ = 33.545, *p* = 0.000) for (**b**) followed by post hoc Tukey’s test.

Previous studies have shown that enhanced Akt-S6K1 signaling may be causal for certain aspects of symptoms in *Fmr1* KO mice^18,35^. We confirmed that, with comparable levels of total Akt (Fig. 6a, b; genotype effect: *F*_1,20_ = 0.460, *p* = 0.505; drug effect: *F*_1,20_ = 1.041, *p* = 0.320; genotype × drug interaction: *F*_1,20_ = 0.407, *p* = 0.531) and S6K1 (Fig. 6d, e; genotype effect: *F*_1,20_ = 0.044, *p* = 0.836; drug effect: *F*_1,20_ = 0.140, *p* = 0.713; genotype × drug interaction: *F*_1,20_ = 0.045, *p* = 0.834), the levels of pAkt (Fig. 6a, c, genotype effect: *F*_1,20_ = 27.793, *p* = 0.000) and pS6K1 (Fig. 6d, f, genotype effect: *F*_1, 20_ = 79.221, *p* = 0.000) are elevated in the hippocampus of *Fmr1* KO mice. Administration of trifluoperazine (0.05 mg/kg) in *Fmr1* KO mice normalized the elevated pAkt (Fig. 6a, c; drug effect: *F*_1,20_ = 25.791, *p* = 0.000; genotype × drug interaction: *F*_1,20_ = 22.512, *p* = 0.000) and pS6K1 (Fig. 6d, f; drug effect: *F*_1,20_ = 40.388, *p* = 0.000; genotype × drug interaction: *F*_1,20_ = 35.893, *p* = 0.000) to the WT level. Together, these results demonstrate that trifluoperazine can normalize the aberrantly elevated PI3K-Akt-S6K1 signaling in *Fmr1* KO neurons. Further, Akt activity (as indicated by pAkt) in *Fmr1* KO neurons is more sensitive to trifluoperazine than in WT neurons (Fig. 7). We found that trifluoperazine at 0.25, 0.5, and 1 μM dampened pAkt in *Fmr1* KO (Fig. 7b) but not in WT neurons (Fig. 7a). Akt activity in WT neurons was significantly suppressed by 2 and 10 μM trifluoperazine (Fig. 7a).

### No effect of D1 and D2 antagonists on behavioral symptoms

To directly test the possibility that therapeutic effect of trifluoperazine is mediated through its activity against dopamine receptors, we used other known D1 and D2 receptor (D1R and D2R) antagonists. At concentrations that affect dopamine receptor function^36–38^, neither the D1R antagonist SCH23390 nor the D2R antagonist raclopride inhibited pAkt in neurons (Supplementary Fig. 10a, b). Intriguingly, we found that the D1R antagonist SCH23390 bi-directionally affects pS6K1. SCH23390 at 1 and 2 μM enhanced pS6K1. At 10 μM, SCH23390 suppressed pS6K1 (Supplementary Fig. 10c). The D2R antagonist raclopride significantly suppressed pS6K1 (Supplementary Fig. 10d), but the degree of suppression was milder than the effect of trifluoperazine (Fig. 5f). It is known that, besides PI3K-Akt, pS6K1 may also be affected by other signaling molecules such as mTOR. We found that the mTOR inhibitor rapamycin had no effect on pAkt but eliminated the pS6K1 signal (Supplementary Fig. 11). These data implicate that the D1R/D2R antagonists have different pharmacological activity from trifluoperazine and mTOR inhibitor.

**Fig. 8:**
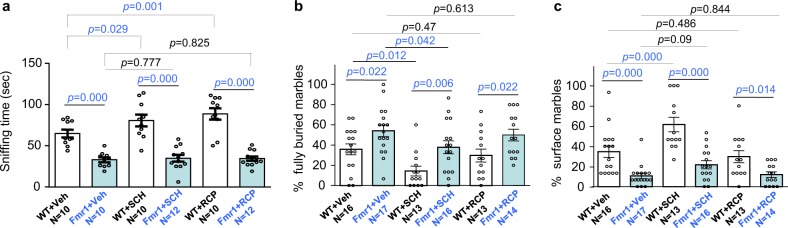
Effects of D1R and D2R antagonists on sociability and repetitive behavior. Wild type (WT) and *Fmr1* KO mice (Fmr1) were i.p. injected with vehicle (Veh), or the D1R antagonist SCH23390 (SCH, 0.05 mg/kg), or the D2R antagonist raclopride (RCP, 0.3 mg/kg). One hour after injection, mice were examined by the 3-chamber social interaction (determined by paradigm 1) (**a**) or marble bury test (**b**, **c**). Direct interaction with the novel stimulus mouse (as indicated by sniffing time) is presented in **a**. For marble bury activity, % of fully buried marble and marbles on the surface is presented in **b**, **c**, respectively. The *p* values were determined by two-way ANOVA followed by Holm-Sidak test.

We further tested the in vivo effects of D1R/D2R antagonists on behavior. In the social interaction test, SCH23390 and raclopride both increased social interaction in WT mice (Fig. 8a; genotype effect: *F*_1,58_ = 121.132, *p* = 0.000; treatment effect: *F*_2,58_ = 3.447, *p* = 0.039; genotype × treatment interaction: *F*_2,58_ = 2.592, *p* = 0.084) (*p* = 0.029 for SCH23390; *p* = 0.001 for raclopride; post hoc Holm-Sidak test). They did not affect *Fmr1* KO mice (*p* = 0.777 for SCH23390, *p* = 0.825 for raclopride; post hoc Holm-Sidak test). In the marble bury test, vehicle-treated and drug-treated *Fmr1* KO mice displayed excessive marble burying activity compared to their corresponding WT groups (for the fully buried marbles in Fig. 8b; genotype effect: *F*_1,83_ = 18.66, *p* = 0.000; drug effect: *F*_2,83_ = 5.637, *p* = 0.005; genotype–drug interaction: *F*_2,83_ = 0.107, *p* = 0.899) (for the surface marbles in Fig. 8c; genotype effect: *F*_1,83_ = 47.509, *p* = 0.000; drug effect: *F*_2,83_ = 11.150, *p* = 0.000; genotype–drug interaction: *F*_2,83_ = 2.660, *p* = 0.076). SCH23390 non-specifically decreased the % of fully buried marbles in both WT and *Fmr1* KO mice (Fig. 8b). For % of surface marbles, SCH23390 had effect on WT but not *Fmr1* KO mice (Fig. 8c). The D2R antagonist raclopride affected neither WT nor *Fmr1* KO mice (Fig. 8b, c). These data show that the D1R/D2R antagonists have different in vivo effects comparing to trifluoperazine, suggesting that the therapeutic effect of trifluoperazine is unlikely due to its inhibitory activity against dopamine receptors.

## Discussion

Compared to de novo therapeutic development, repurposing FDA-approved drugs offers several benefits including reduction of cost and potentially sooner clinical application. Regarding examination of FDA-approved drugs for FXS treatment, recent studies mainly focused on the known disease mechanism and used hypothesis-driven approaches. For instance, therapeutic testing with metformin and lovastatin, which show promising efficacy in both FXS mouse model^19,39^ and small scale open-label human studies^40,41^, is theoretically based on their pharmacological actions against mTORC1 and ERK1/2 (extracellular signal-regulated kinase 1/2), the activity of which is abnormally elevated in FXS mouse model and patients^3^. In this study, we used whole transcriptome analysis as an unbiased approach to identify potential therapeutics. Gene signature analysis with normal neurons and psychiatric disorder-affected specimens suggests an intriguing possibility that transcriptome landscape and cell function are associated and may mutually affect each other. Related to disease mechanism, genomic alteration is considered as an emerging molecular phenotype and pathological endpoint. Intriguingly, recent computational comparison of gene signatures associated with psychiatric disorders and drug treatment suggests an attractive but yet to-be-validated approach for therapy development and drug repurposing^7,8^. Here, we compared transcriptome alterations caused by *Fmr1* deficiency and CMAP drugs. The computational analysis identified trifluoperazine as a potential therapeutic. It is important to note that transcriptome data in the CMAP database were collected from cancer cell lines rather than neurons. Interestingly, we were able to validate the CMAP-predicted effect of trifluoperazine in neurons and FXS mouse model. Two recent computational studies also found that the CMAP data from cancer cells may be effectively used to predict therapy or mechanism for neurological disorders^7,8^.

Trifluoperazine, belonging to the phenothiazine group, is a well-known antipsychotic. Interestingly, some antipsychotics such as haloperidol, risperidone, and aripiprazole have been noted to alleviate irritability and aggression in autism and FXS patients^42–44^. The main side effects include extrapyramidal symptoms (EPS) and sedation. In particular for trifluoperazine, long-term use in the 6–40 mg/day range exerts EPS^16^. However, in theory, side effects depend on drug dose, and human studies demonstrate that EPS can be successfully managed through dose adjustment. Here, we observed robust efficacy of low dose trifluoperazine at 0.05 mg/kg, which, according to the well-accepted FDA formula^45^, is equivalent to 0.004 mg/kg for human (or 0.24 mg for a person with 60 kg body weight). This dose is highly applicable and tolerated, as it is significantly lower than 5 mg/day, which, judged by the Extrapyramidal Symptom Rating Scale, does not cause adverse side effects in humans^17^. Notably, low dose trifluoperazine (i.e., 1 mg, which is about 4× higher than 0.24 mg) is also used in combination with antidepressant in human patients, and sold under the brand names of Parmodalin and Jatrosom N.

The primary application of trifluoperazine in schizophrenia patients shows strong effects on correcting the positive symptoms possibly through its inhibition on dopamine receptors. It is known that the anti-dopamine effects of trifluoperazine decrease locomotor activity but does not improve cognitive function. We found that trifluoperazine at 0.05 mg/kg does not affect locomotor activity in WT mice in the light-dark and open field test. In *Fmr1* KO mice, trifluoperazine normalizes the excessive locomotor transition in the light-dark test but not in the open field paradigm. Notably, 0.05 mg/kg trifluoperazine rescued the cognitive impairment in *Fmr1* KO mice. Interestingly, certain aspects of FXS-associated abnormality are caused by decreased dopamine function and dopamine agonist corrects certain phenotypes in FXS mouse^29^. We further examined the effect of other known D1R and D2R antagonists, and found no therapeutic efficacy in correcting FXS-associated abnormalities. These lines of evidence suggest that the therapeutic effects of low dose trifluoperazine are unlikely due to its anti-dopamine activity.

Our study further used computational screening with CMAP to identify a potential pharmacological mechanism underlying the therapeutic effects of trifluoperazine. Intriguingly, although gene signature induced by trifluoperazine shows similarity to those induced by the known HDAC and PI3K inhibitors, we found trifluoperazine effect on PI3K activity but not histone acetylation. However, as our assay only examined acetylation of H3 and H4 at specific lysine residuals (i.e., K9 of H3 and K8 of H4), more exhaustive investigation may be needed to totally exclude potential trifluoperazine activity against HDAC. Since all available small molecule drugs have promiscuous pharmacological targets, we do not exclude the possibility that the therapeutic effects of trifluoperazine are mediated by regulating other signaling pathways in addition to the inhibition of the PI3K-Akt-S6K1 cascade. Nevertheless, our results do suggest that drugs with similar effects on transcriptome signature may have similar MOA (mechanism of action) and warrant experimental validation.

Notably, some of the top-ranked trifluoperazine similarity compounds also induce transcriptome alterations that are oppositional to that caused by *Fmr1* deficiency. For example, gene signatures of trichostatin A (in PC3 cell, ranked at #6) and thioridazine (in PC3 cell, ranked at #2) show positive similarity to trifluoperazine signature (Table 1). They show negative similarity to *Fmr1* KO neuron signature, and are ranked at #1 and #9, respectively (Fig. 1d). Although these compounds are either not approved by FDA or withdrawn from clinical use, it would be interesting to test whether they can correct certain pathological aspects of FXS in animal models. Along the same line, our results advocate future studies to repurpose other CMAP-identified trifluoperazine similarity drugs (Supplementary Data 7).

Intriguingly, our unbiased computational prediction of trifluoperazine efficacy and its action against PI3K activity are strikingly coincident with a previously suggested disease mechanism. Recent studies have found that, in the absence of FMRP, expression levels of PI3K activator PIKE and p110ß subunit are elevated in *Fmr1* deficient cells^27,31^, leading to elevated PI3K activity and over-activation of the Akt-S6K1 signaling cascade^18,26^. Therapeutic value of correcting the altered PI3K-Akt-S6K1 signaling in FXS is strongly suggested by that genetic removal/reduction of S6K1, PIKE, and p110ß subunit of PI3K can rescue the elevated overall protein synthesis and multiple behavioral abnormalities in *Fmr1* KO mice^18,28,35^. A more recent study showed that systemic injection of a novel p110β-specific inhibitor GSK2702926A, a similarly structured compound of which is under investigation in a cancer clinical trial, rescues cellular and behavioral abnormalities in *Fmr1* KO mice^46^. To realize therapeutic value of targeting the PI3K-Akt-S6K1 signaling cascade, a significant advancement is to find a clinically suitable approach to effectively dampen PI3K-Akt-S6K1 activity in FXS. By using the FDA-approved trifluoperazine, we demonstrate that acute administration of a drug with PI3K inhibiting activity is sufficient to rescue multiple aspects of FXS-associated pathology, supporting the practical value of targeting PI3K-Akt-S6K1 signaling. Considering that human FXS samples also display elevated PI3K-Akt-S6K1 signaling^26,30^, our data encourage future investigation to determine the efficacy of trifluoperazine in human patients.

We acknowledge that trifluoperazine does not correct all FXS-associated symptoms that were examined in this study. Lack of absolute efficacy is common for drugs used for complex neurological disorders. It is noticed that targeting ERK1/2 or PI3K by lovastatin, metformin, and GSK2702926A rescues some but not all symptoms^19,39,46^. Such limitation may be due to multiple pathological alterations in FXS; combinatorial targeting multiple disease factors represents an emerging idea to achieve more robust therapeutic outcome^47^. Alternatively, adjustment of dose and treatment duration may also be considered for future studies. Furthermore, although FXS is a main cause for autism and intellectually disability (ID), we do not expect that trifluoperazine would show general therapeutic effects in other autism and ID models.

We acknowledge that FDA has just approved the first PI3K inhibitor idelalisib in July 2014 for the treatment of leukemia. Idelalisib (not included in CMAP database) shows high specificity against the p110δ isoform of PI3K, which is expressed mainly in hemocyte and marginally in limited brain regions (according to Allen Brain Atlas). Considering that the dysfunction in FXS involves numerous brain structures, the therapeutic efficacy of idelalisib may be limited. Another complication is that its pharmacokinetic profile has only been examined for blood absorption and excretion. Whether idelalisib can cross the blood brain barrier has not been determined and not known^48^.

In summary, our study supports the value of holistic transcriptome analysis in therapeutic discovery and repurposing FDA-approved drugs for FXS treatment. Experimental validation of trifluoperazine efficacy in FXS mouse model suggests a practical treatment strategy. Our results along with a recent computational study^8^ also encourage broader application of transcriptome screening with public databases in guiding drug discovery for other neurological disorders.

## Methods

### Animals

Male *Fmr1* knockout (KO) mice and their wild type (WT) littermates on C57BL/6 background were obtained from breeding pairs consisting of heterozygous female and WT male mouse. Animals were housed in the Campus Animal Research facility. The Institutional Animal Care and Use Committee approved all procedures. The mice had ad libitum access to water and food and were housed under 12 h dark/light cycle.

### Analysis of transcriptome in *Fmr1* KO neurons

Whole genome transcript changes were determined by RNA-seq with total RNA extracted from primary hippocampal cultures at DIV (days in vitro) 14. Triplicate samples/repeats were analyzed and compared between wild type (WT) and *Fmr1* KO genotype. Total RNA was extracted by the TRIzol method (Invitrogen) followed by purification with RNAeasy kit from Qiagen. Purified total RNA with RIN > 8 was subjected to RNA-seq. Raw data were processed through adapter cleaning (with Fastx Clipper), trimming (with sickle, https://github.com/najoshi/sickle), junk sequence removal, mapping clean reads to the mouse reference genome (version: GRCm38) using TopHat2, and gene construction with Cufflinks^49^. Differentially expressed genes (DEGs) between WT and *Fmr1* KO samples were assessed with Cuffdiff. The resulting DEGs were ranked based on expression differences. GO (gene ontology) enrichment and KEGG (Kyoto Encyclopedia of Genes and Genomes) pathways were identified with DAVID (Database for Annotation, Visualization and Integrated Discovery; http://david.abcc.ncifcrf.gov/home.jsp)^50^.

### Computational prediction on drug effect with CMAP

Transcriptome alterations in *Fmr1* KO neurons, including upregulated and downregulated DEGs (differentially expressed genes) were compared with gene signatures in CMAP database (http://www.broadintitute.org/cmap/)^9^. DEGs were first converted to corresponding human gene probes using Batch Query at NetAffx (http://www.affymetrix.com/analysis/netaffx/index.affx). The detailed list of signature probes for DEGs in *Fmr1* KO neurons is shown in Supplementary Data 8.

To obtain gene expression signature for trifluoperazine, raw microarray data sets in PC3 cells treated with trifluoperazine and corresponding vehicle control were downloaded from CMAP database. The raw data were normalized using robust multi-array average algorithm (RMA)^51^ from the R package affy (v1.44.0)^52^ (https://www.bioconductor.org/about/). Genes were then ranked based on the expression differences between the drug-treated and vehicle controls. The signature genes were selected using “up threshold” and “down threshold” as defined by CMAP. The detailed list of signature probes for trifluoperazine is shown in Supplementary Data 6.

Gene signature of *Fmr1* KO neuron or trifluoperazine was uploaded to CMAP quick query page to search for compounds that induce similar and oppositional transcriptome changes. CMAP has devised a tool to compute enrichment of upregulated and downregulated genes from transcriptome changes in compound/drug-treated cells, and the results for all instances related to a specific compound are combined to form “connectivity score” (i.e., similarity mean) for each compound^9^. After executing a query, the top hit chemical list was returned, displaying each compound’s associated similarity mean, number of arrays, enrichment, and *p* value.

### In vivo drug administration

Trifluoperazine dihydrochloride (Sigma-Aldrich), SCH23390 (Sigma-Aldrich), and raclopride (Sigma-Aldrich) solution in water was i.p. injected into mice at 0.05 mg/kg, 0.05 mg/kg, and 0.3 mg/kg, respectively. For passive avoidance, trifluoperazine was administered 1 h before or after training. For all other behavioral paradigms, drug was administered 1 h before examination. Control mice were treated similarly but injected with vehicle.

To test the effect of repeated drug administration, trifluoperazine at 0.05 mg/kg was injected once per day for 8 days. One hour after the last injection, mice were examined by behavior tests.

### Behavioral tests

To examine sociability, two different behavioral paradigms that have been used to detect social interaction deficits in *Fmr1* KO mice^18,19^ were performed. For the first paradigm, 2-month to 3-month-old mouse was placed in the center chamber of a 3-chamber social interaction box. Mouse was allowed to freely explore all 3 chambers for 5 min. Mouse showing significant preference for a particular chamber was not used for further testing. Following the exploration, mouse was momentarily restricted (for less than 10 s) in the center chamber with the entry doors to the side chamber closed. The 10-min sociability test started with placement of a novel stimulus mouse in a wire enclosure and an empty wire enclosure, respectively, in one of the side chambers, followed by opening the chamber-connecting doors. For the second paradigm, similar procedure was performed except for that an un-animated object was placed under the wire enclosure in the non-social chamber. As a social interaction index, total time spent in sniffing the novel stimulus mouse enclosure was recorded.

To perform the marble-burying test, 2-month to 3-month old mice were placed in a box (27 cm by 15 cm box with 12-cm high walls) with 7.5 cm depth of bedding for 1 h prior to the test. The mouse was then briefly removed from the testing box and 15 marbles were evenly arranged in a 5 by 3 pattern on the surface of the bedding. The mouse was reintroduced into the testing box and was allowed to bury marbles for 10 min. At the end of the testing period, the mouse was removed from the box and the number of marbles that were fully buried, partially buried, and left on the surface was counted.

To determine stereotypic digging, 2.5-month to 3.5-month-old mice were introduced to fresh mouse cage with 1-inch bedding material. During the 5-min examination, latency to the first digging episode, total number of digging episodes, and accumulative time spent in digging were recorded.

To determine stereotypic self-grooming, 2.5-month to 3.5-month-old mice were placed in a plexiglas cage with 1 cm fresh bedding without nesting material. After a 10-min habituation period, the number of self-grooming episode and duration were scored for 10 min^53^.

To examine animal activity in light-dark test, 2.5-month to 3.5-month-old mice were placed in the dark half of the light-dark chamber and the trap door was opened 1 min later. The mice were allowed to move freely between the dark and the lit chambers for 5 min. Time spent in the lit chamber and number of crossing into the lit side were recorded.

To determine animal activity in an open field arena, 2-month to 3-month-old mice were placed in the center of an open field chamber, and were allowed to move freely for 1 h. Ambulatory movement distance in the whole arena and in the center area of the open field were determined every 10-min during the 1-h testing period by the TruScan Photo Beam Activity System (Coulbourn Instruments, Whitehall, PA).

To examine passive avoidance learning, 2.5-month to 3.5-month-old mice were introduced into the lit half of the passive avoidance chamber (Coulbourn Instruments, Whitehall, PA) and allowed to explore for 1 min before the trap door was opened. The trap door was closed as soon as the mouse entered the dark chamber. A mild foot shock (0.7 mA for 2 s) was immediately delivered. The mouse was removed from the dark chamber and returned to its home cage 30 s after the delivery of foot shock. The mice were tested 24 h after training. During testing, the mouse was put in the lit chamber and crossover latency to the dark chamber was recorded. If mice stayed in the lit chamber for more than 600 s, they were manually removed from the chamber, and 600 s was used as their crossover latency. In a stronger training paradigm, three electric foot shocks (0.7 mA for 2 s per shock with 10 s interval) were delivered during training when animal entered the dark chamber.

To measure audiogenic seizures (AGS), 21-days to 24-days-old mice were placed in a box (30 cm L by 17 cm W by 12 cm H) with a flat plastic lid. A personal alarm (from Streetwise, item # SWPDAL) was taped to the lid of the box and wired to a DC power supply to keep the sound amplitude constant. The mouse was allowed to acclimatize to the box for 5 min, following which a 120-dB sound was emitted from the alarm for 2 min. The number of mice undergoing seizure within the 2-min period was counted. Audiogenic seizures were considered when wild running and/or clonic/tonic seizures occur.

Some of the animals were re-used in different behavioral tests (see Supplementary Fig. 12). Mice used for open field test were later used for social interaction. Some mice used for marble-burying test were later used for light-dark test. Mice used for stereotypic digging were used later for passive avoidance. The interval between the two behavioral tests was at least 10 days.

### Neuronal culture and measurements of protein synthesis

Primary hippocampal neurons were cultured with samples obtained from postnatal day 0 WT and *Fmr1* KO mice. Protein synthesis was determined by the SUnSET method^18,25^. DIV 14 hippocampal neurons were pre-treated with trifluoperazine at various concentrations (0.5, 1, 2, 10, and 20 μM as indicated) for 30 min followed by 5 μg/ml puromycin (Sigma, Cat #P8833) treatment for 30 min. Cells were lysed in Buffer H (50 mM β-glycerophosphate, 1.5 mM EGTA, 0.1 mM Na_3_VO_4_, 1 mM DTT). The samples were sonicated and centrifuged. An aliquot of the supernatant was used to determine protein concentration, and the rest was denatured in Laemmli buffer. Twenty microgram protein was separated by 4–20% SDS-PAGE (Invitrogen) and transferred onto nitrocellulose membranes. The membranes were probed with anti-puromycin antibody (KeraFAST, Cat # EQ0001, 1:1000). The relative amount of loading was determined by β-actin. ImageJ was used to measure the combined signal intensity of proteins with molecular weights ranging from 15 to 250 kDa.

### Examination of drug effects by Western blot

To examine the in vitro effects, DIV 14 hippocampal neurons (WT and *Fmr1* KO as indicated) were treated with vehicle, vorinostat (20 μM), trichostatin A (20 μM), LY-294002 (20 µM), trifluoperazine (at 0.25, 0.5, 1, 2, 10, and 20 μM as indicated), SCH23390 (at 1, 2, and 10 μM as indicated), raclopride (at 1, 2, and 10 μM as indicated), and rapamycin (20 nM) for 1 h. Immediately after treatment, neurons were lysed in Laemmli buffer (62.5 mM Tris-HCL, pH 6.8, 10% glycerol, 2% SDS, 5% 2-mercaptoethanol, 0.005% bromophenol blue). To examine the in vivo effect, 2-month to-3-month old wild type (WT) and *Fmr1* KO mice were injected with 0.05 mg/kg trifluoperazine or vehicle. Hippocampus was rapidly dissected 1 h after injection and homogenized in RIPA buffer (50 mM Tris-HCL, pH 7.6, 150 mM NaCl, 1 mM EDTA, 0.25% sodium deoxycholate, 0.5% NP-40). Protein content in hippocampal homogenates was determined using Bradford’s assay. Same amount of cell extract/lysate was separated by SDS-PAGE followed by transferring to nitrocellulose membranes. The following antibodies were used to detect the corresponding targets. Anti-S6K1 (Cat #2708, 1:1000 dilution), anti-phospho-S6K1 (at Thr389) (Cat #9234, 1:1000 dilution), anti-phospho-Akt (at Ser473) (Cat #4060, 1:1000 dilution), anti-Akt (Cat #9272, 1:1000 dilution), anti-acetylated histone H3 at K9 (Cat #9649P, 1:1000 dilution), anti-acetylated histone H4 at K8 (Cat #2594P, 1:1000 dilution), anti-histone H3 (Cat #4499 P, 1:1000 dilution), and anti-histone H4 (Cat #13919 P, 1:1000 dilution) were from Cell Signaling. β-actin antibody was from Sigma (Cat #A5441, 1:10,000 dilution). Anti-FMRP (mouse monoclonal antibody 2F5, 1:1000) was a generous gift from Dr. Jennifer Darnell at the Rockefeller University. For quantification purpose, level of phospho-S6K1, phospho-Akt, acetylated-H3, and acetylated-H4 was normalized to the level of total S6K1, Akt, H3, and H4, respectively. The membrane was normally first blotted for the phosphorylated and acetylated proteins. Then, the same membrane was stripped; the effectiveness of stripping/removing the phospho-specific or acetylation-specific antibodies was checked by blotting with secondary antibody alone. The successfully stripped membrane was then washed and re-probed with antibodies against the corresponding total protein. The Western blot signal was detected by the Odyssey digital imaging system. Relative intensity of the Western blot signal in the no treatment control group, which was quantified using ImageJ (NIH, MD, USA), was defined as 1. Signal in the treatment samples was compared to the control group.

### PI3 kinase assay

Hippocampal neurons (cultured from WT mice) on DIV 14 were treated with trifluoperazine (20 μM) or vehicle for 30 min. Neurons were then washed once with PBS and lysed in 100 μl lysis buffer (50 mM Tris pH 7.4, 40 mM NaCl, 1 mM EDTA, 0.5% Triton, 1.5 mM Na_3_VO_4_, 50 mM NaF, 10 mM sodium pyrophosphate) with proteinase inhibitor (Roche) on ice for 10 min. Lysates were cleared by centrifugation and protein concentration was determined by Bradford method. PI3 kinase activity was measured using PI3 kinase activity ELISA kit from Echelon Biosciences according to the manufacture’s protocol. Briefly, 30 μl of lysate was incubated with 30 μl of 10 μM PIP2 and 20 μM trifluoperazine or vehicle at 37 °C for 3 h. The reaction buffer contained 2 mM DTT and 100 μM ATP. The amount of PIP3 produced by PI3 kinase was determined by ELISA. The relative amounts of PIP3 in the samples were used to determine the activity of PI3 kinase.

### Examination of neuron viability

DIV 14 hippocampal neurons were treated with vehicle or 10 or 20 μM trifluoperazine for 1 h. As a positive control, NMDA treatment (50 μM for 30 min) was used to trigger neuronal cell death. Immediately after treatment, drugs were removed by replacement with conditioned medium. Twenty hours after treatment, neurons were fixed with 4% formaldehyde and stained with DAPI. As described in our previous study^54^, neuron survival (i.e., viability) was quantified by the ratio of neurons showing diffused nuclear DAPI staining to neurons showing condensed nuclear DAPI staining.

### Statistics and reproducibility

Animals subjected to different treatment were randomly assigned. Sample size was estimated by power analysis (80% power and probability level of 0.05 along with the anticipated effect size) or based on previous reports. Data were collected from two (for biochemical analyses with primary neurons) or more than two litters (for behavioral analyses and in vivo biochemical analysis) and combined. Experimenters were blind to the treatments and genotypes, which were decoded before data analyses. Data with normal distribution were analyzed by Student’s *t*-test or ANOVA. Student’s *t*-test (two-sided) was used to compare data collected from two groups. One-way ANOVA and Tukey’s post hoc tests were used to analyze data involving different treatments. Two-way ANOVA and Holm-Sidak post hoc tests were to analyze data involving genotype effect and treatment effect. Three-way repeated measures ANOVA was used to analyze the open field data at different time points during the 60-min test. When normal distribution and equal variance were not detected (i.e., crossover latency during testing for passive avoidance), the numerical values were first converted to categorical data (latency of 600 s or less than 600 s was assigned to different category) and then analyzed by Fisher’s exact test. Data reporting AGS were analyzed by Chi-square and/or Fisher’s exact test. Data are expressed as mean ± SEM. Differences with *p* value less than 0.05 were considered significant. SPSS 11.5 for Windows (IBM) was used for all data analysis with consideration of adjustment for multiple conparisons.

The statistical method for CMAP analysis was described previously^9^. In brief, the *p* value was computed using Kolmogorov-Smirnov statistics. It is estimated empirically by computing the enrichment of 100,000 sets of instances randomly selected from all instances in the result.

### Reporting summary

Further information on research design is available in the Nature Research Reporting Summary linked to this article.

## Supplementary information

Supplementary Information

Description of Additional Supplementary Files

Supplementary Data 1

Supplementary Data 2

Supplementary Data 3

Supplementary Data 4

Supplementary Data 5

Supplementary Data 6

Supplementary Data 7

Supplementary Data 8

Supplementary Data 9

Supplementary Data 10

Peer Review File

Reporting Summary

## Data Availability

RNA-seq data have been deposited and are available in Gene Expression Omnibus under accession number GSE114015. All individual data point is presented in graphs. Source data used for graphs are provided in Supplementary Data 9 and 10. The full gel Western blot images are provided in Supplementary Figs. 13 and 14. All data associated with this study are available from the corresponding author upon reasonable request.
