# Supplementary material for: Transcriptome signature analysis repurposes trifluoperazine for the treatment of fragile X syndrome in mouse model
Source: Commun Biol. 2020 Mar 16;3:127. doi: 10.1038/s42003-020-0833-4 (PMC7075969; doi:10.1038/s42003-020-0833-4)
Supplement: Supplementary file 1 — Supplementary Information [file 42003_2020_833_MOESM1_ESM.pdf]

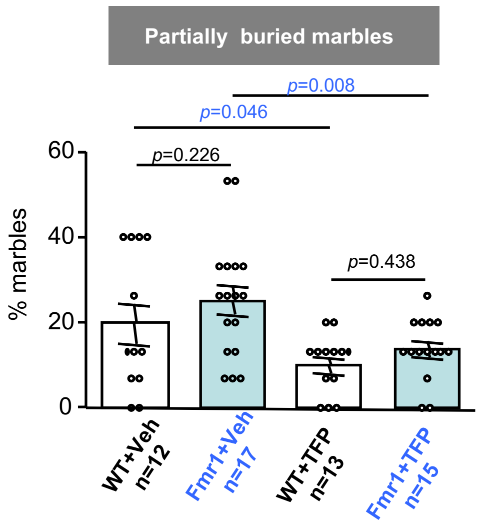

Supplementary Figure 1 Trifluoperazine (TFP) non-specifically decreases partially buried marbles in wild type (WT) and *Fmr1* KO (Fmr1) mice. Veh: vehicle. The p values between the indicated 2 groups were determined by two-way ANOVA followed by Holm-Sidak test.

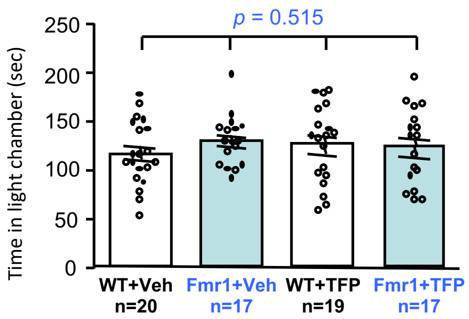

Supplementary Figure 2 Wild type (WT) and *Fmr1* KO (Fmr1) mice spent similar time in the lit chamber during the light-dark test. Vehicle (Veh)- or trifluoperazine (TFP)-injected mice were subjected to light-dark test for 5 min. Time spent in the lit chamber was scored. The p value was determined by two-way ANOVA.

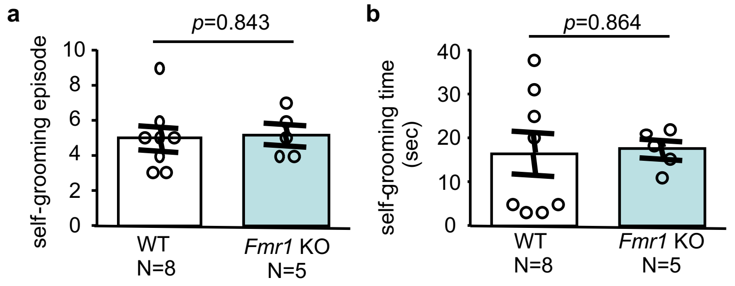

Supplementary Figure 3 Wild type (WT) and *Fmr1* knockout (KO) mice show comparable self-grooming. WT and KO mice were examined for self-grooming. The number of grooming session (i.e. episode) is presented in **a**. Total grooming time is presented in **b**. The p values were determined by Student's t-test.

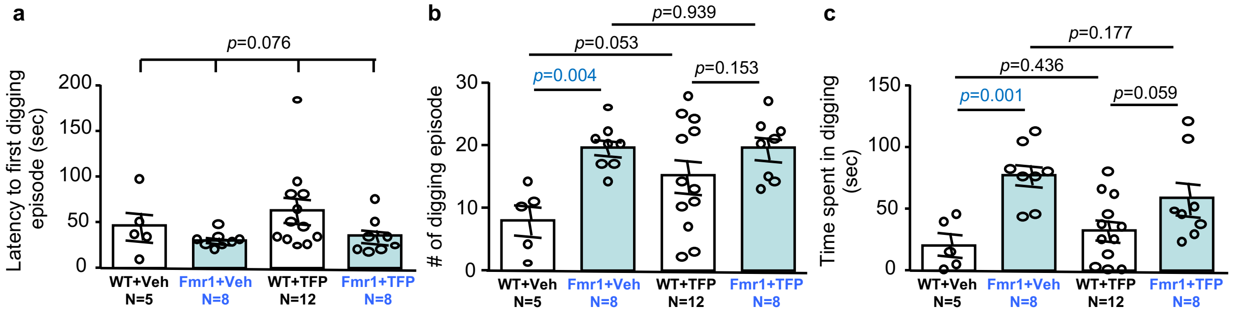

Supplementary Figure 4 Trifluoperazine (TFP) does not significantly affect stereotypic digging behavior. (**a**) Wild type (WT) and *Fmr1* KO (Fmr1) mice show comparable latency to the first digging behavior, and are not affected by TFP. Genotype effect: $F_{1, 29} = 3.395$, $p = 0.076$; drug effect: $F_{1, 29} = 0.783$, $p = 0.383$; genotype-drug interaction: $F_{1, 29} = 0.305$, $p = 0.585$. (**b**) *Fmr1* KO mice show more digging episode, and are not affected by TFP. Genotype effect: $F_{1, 29} = 11.264$, $p = 0.002$; drug effect: $F_{1, 29} = 2.005$, $p = 0.167$; genotype-drug interaction: $F_{1, 29} = 2.313$, $p = 0.139$. (**c**) *Fmr1* KO mice show more total digging time, and are not affected by TFP. Genotype effect: $F_{1, 29} = 15.809$, $p = 0.000$; drug effect: $F_{1, 29} = 0.138$, $p = 0.713$; genotype-drug interaction: $F_{1, 29} = 2.319$, $p = 0.139$. Veh: vehicle. The p values between the indicated 2 groups were determined by two-way ANOVA followed by Holm-Sidak test.

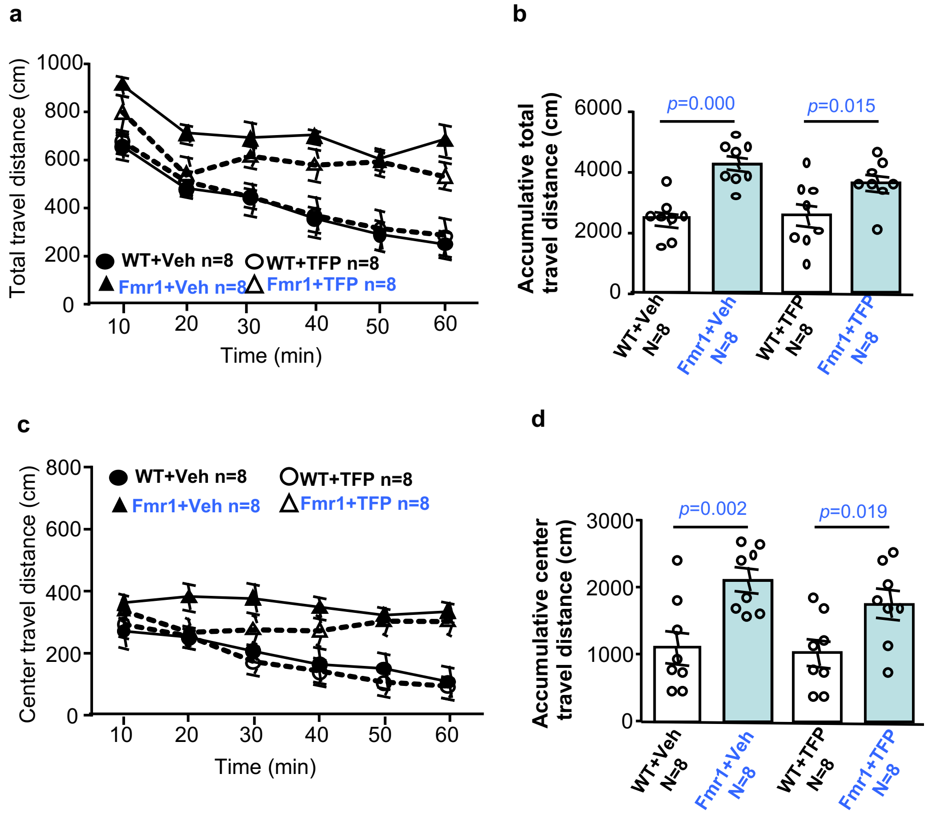

Supplementary Figure 5 Trifluoperazine (TFP) has no effect on locomotor activity in open field. One hour after trifluoperazine administration, mice were subjected to an open field test. During the 1-hour test, ambulatory travel distance in the whole arena (**a** and **b**) or in the center area (**c** and **d**) for each of the 10-min bin (**a** and **c**) and during the whole 1-hour testing (i.e. accumulative travel distance in **b** and **d**) are presented. Veh: vehicle. The p values between the indicated 2 groups were determined by two-way ANOVA followed by Holm-Sidak test.

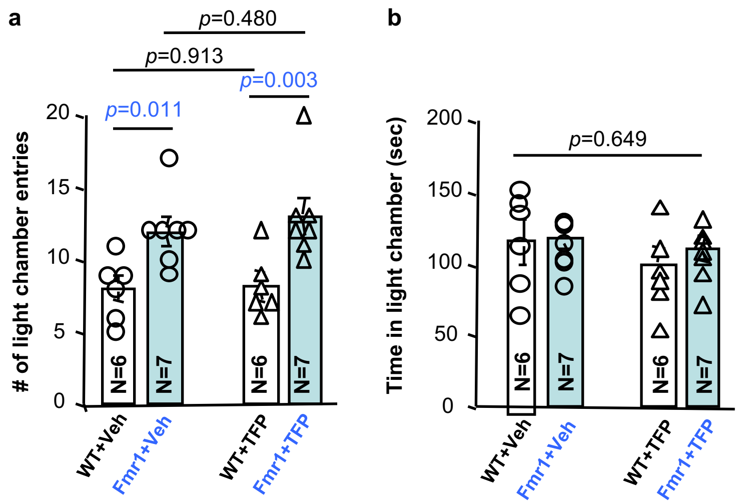

Supplementary Figure 6 The effect of trifluoperazine on locomotion does not persist beyond 24 hours after drug administration. Wild type (WT) and *Fmr1* KO (Fmr1) mice were injected with trifluoperazine (TFP) (0.05 mg/kg) or vehicle (Veh). 24 hours later, these four groups of mice were subjected to light-dark test. **a**. The number of locomotive transition between the lit and dark chamber. The p values between the indicated 2 groups were determined by two-way ANOVA followed by Holm-Sidak test. **b.** Total time stayed in the lit chamber. Genotype effect: $F_{1,22} = 0.213$, $p = 0.649$; drug effect: $F_{1,22} = 1.307$, $p = 0.265$; genotype X drug interaction: $F_{1,22} = 0.552$, $p = 0.466$; determined by two-way ANOVA.

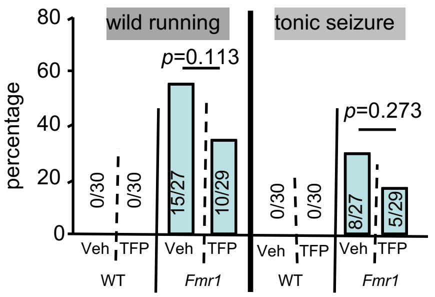

Supplementary Figure 7 Trifluoperazine does not significantly affect audiogenic seizures. Audiogenic seizures were tested with 21- to 24-day old mice. The percentage of animals showing wild running and seizures is presented for wild type (WT) and *Fmr1* KO mice (*Fmr1*) receiving vehicle (Veh) or trifluoperazine (TFP). The p values were determined by Chi-square analysis.

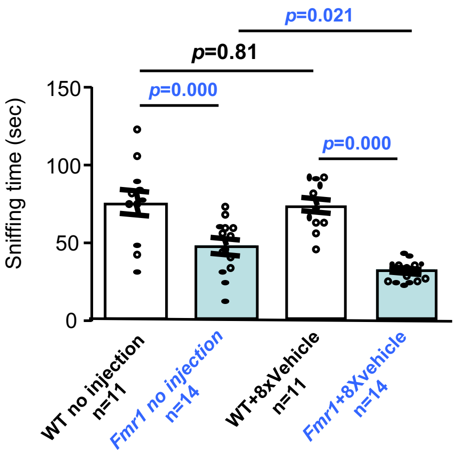

Supplementary Figure 8 The repeated injection procedure reduces sociability in *Fmr1* KO mice. Wild type (WT) and *Fmr1* KO (Fmr1) mice receiving no injection or 8 daily injection of vehicle were examined in the 3-chamber social interaction test (determined by paradigm 1). Time spent in sniffing the novel stimulus mouse enclosure was compared. Regardless of the injection procedure, WT mice show higher sociability than *Fmr1* KO mice (genotype effect: $F_{1,\,46}$ = 49.614, p = 0.000; injection effect: $F_{1,\,46}$ = 3.117, p = 0.084; genotype X injection interaction: $F_{1,\,46}$ = 1.971, p = 0.167). The p values (as shown in the figure) between 2 groups were determined by two-way ANOVA followed by *post-hoc* Holm-Sidak test.

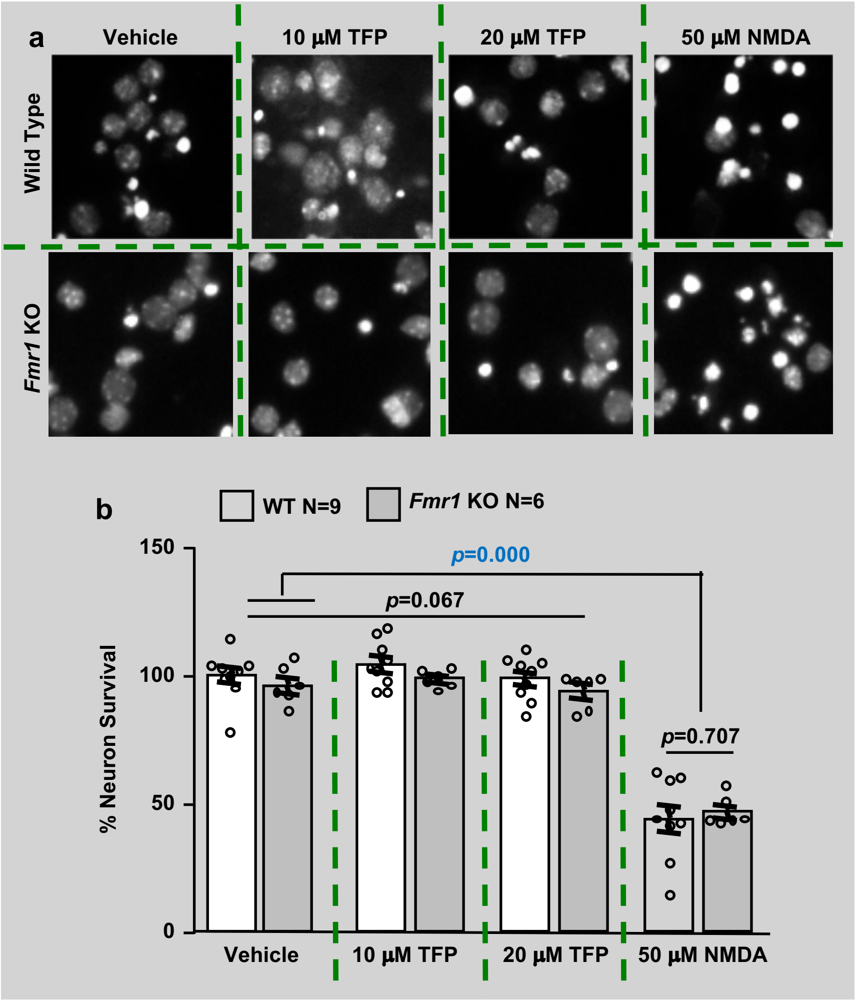

Supplementary Figure 9 Trifluoperazine (TFP) does not affect neuron viability. DIV 14 wild type (WT) and *Fmr1* KO hippocampal neurons were treated with vehicle, 10 μM TFP, 20 μM TFP, or 50 μM NMDA. Representative DAPI staining is shown in **a**. Quantification of viability is shown in **b**. TFP does not cause neuronal cell death (the p value between vehicle and TFP-treated groups was determined by two-way ANOVA). When neurons were treated by NMDA, both WT and *Fmr1* KO neurons showed significant cell death (the p values were determined by two-way ANOVA followed by Holm-Sidak test).

Supplementary Figure 10 Effects of D1 and D2 receptor antagonists on Akt and S6K1 activity. Wild type DIV 14 hippocampal neurons were treated with SCH23390 (**a** and **c**) or raclopride (**b** and **d**) at various concentrations (as indicated) for 1 hour. The levels of pAkt, total Akt, pS6K1, total S6K1, and β-actin were determined by Western blot. The level of pAkt (**a** and **b**) and pS6K1 (**c** and **d**) are normalized to the level of total Akt and total S6K1, respectively. The relative level of pAkt and pS6K1 in the vehicle-treated control group is defined as 1, and all samples are normalized to this group. For **a:** $F_{3, 8} = 3.432$, $p = 0.072$, determined by one-way ANOVA. For **b:** $F_{3, 8} = 1.343$, $p = 0.327$, determined by one-way ANOVA. For **c** and **d**, there were treatment effects: $F_{3, 8} = 32.228$, $p = 0.000$ for **c;** $F_{3, 8} = 14.280$, $p = 0.001$ for **d**. The p values between the treated and control samples in **c** and **d** were determined by one-way ANOVA followed by Tukey's test.

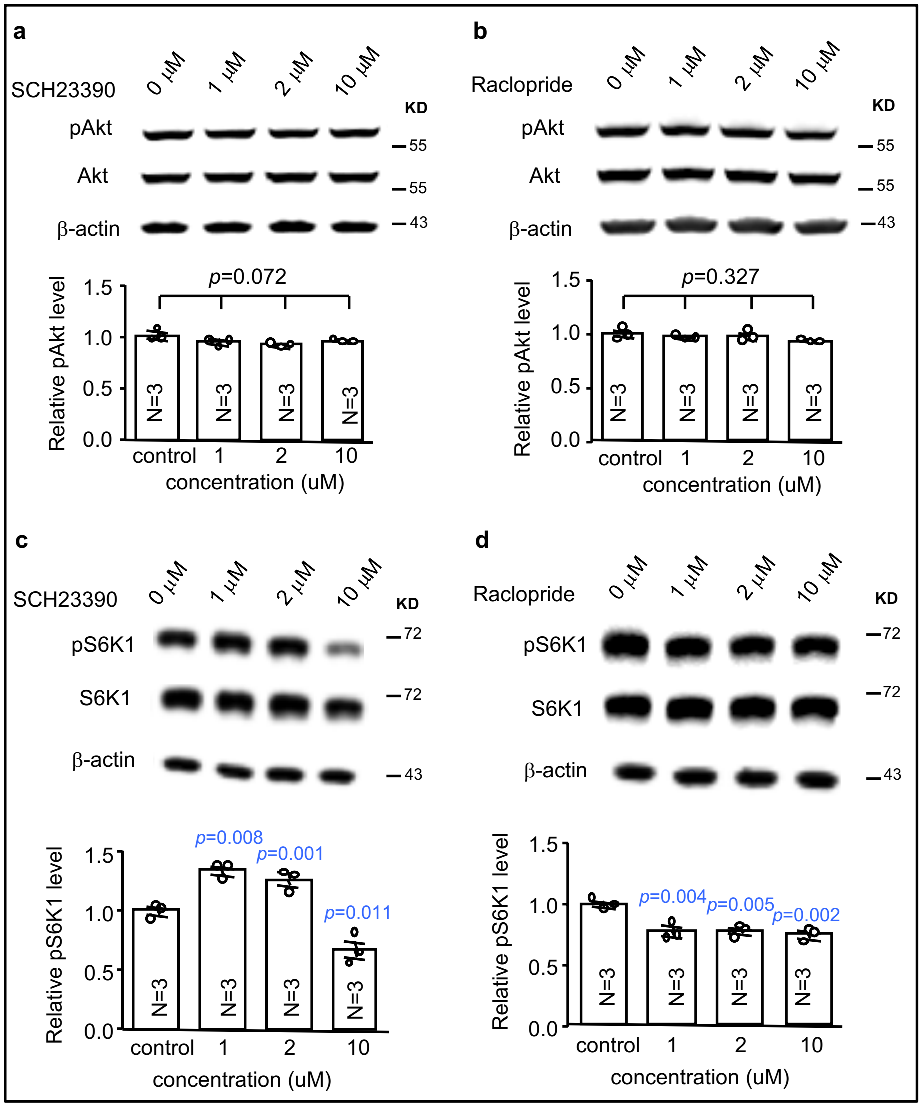

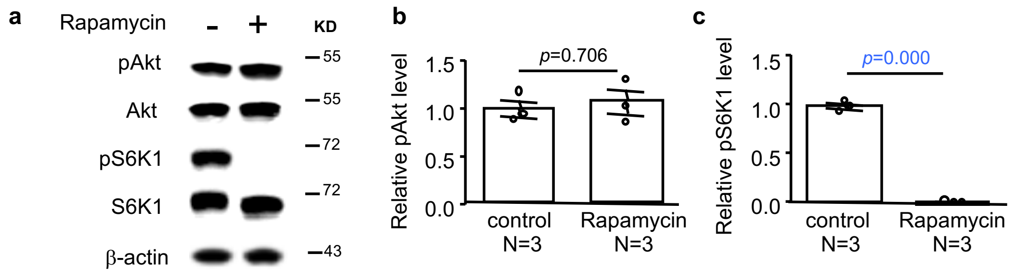

Supplementary Figure 11 Effects of rapamycin on Akt and S6K1 activity. Wild type IV 14 hippocampal neurons were treated with rapamycin (20 nM) for 1 hour. The levels of pAkt, total Akt, pS6K1, total S6K1, and β-actin were determined by Western blot. The level of pAkt (**a** and **b**) and pS6K1 (**a** and **c**) are normalized to the level of total Akt and total S6K1, respectively. The relative level of pAkt and pS6K1 in the vehicle-treated control group is defined as 1, and the rapamycin-treated samples are normalized to the control group. The p values were determined by Student's t-test.

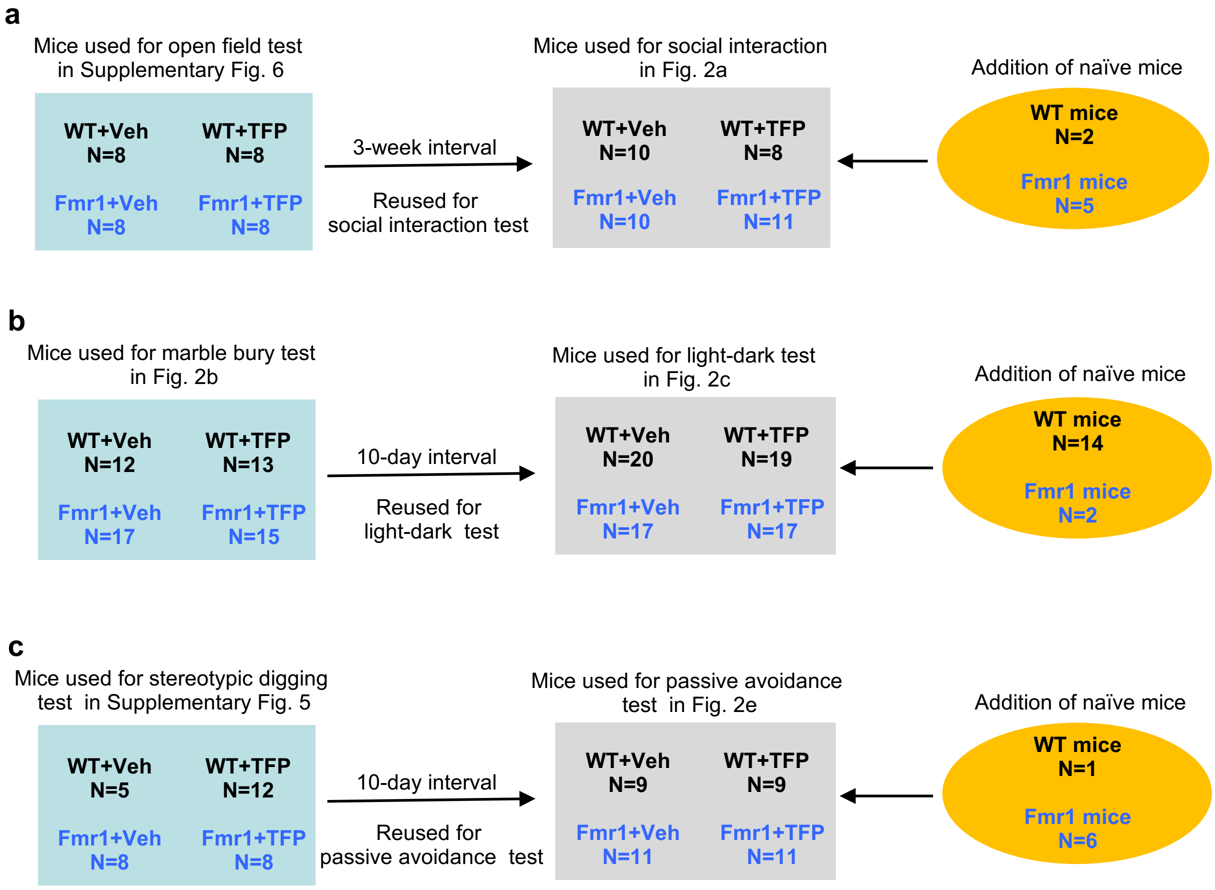

Supplementary Figure 12 Animals that are reused for different behavioral tests. a. Wild type (WT) and *Fmr1* knockout (Fmr1) mice were first used for open field test, and then reused for social interaction test with the addition of naïve mice. **b.** WT and *Fmr1* mice were first used for marble bury test, and then reused for light-dark test with the addition of naïve mice. **c.** WT and *Fmr1* mice were first used for stereotypic digging test, and then reused for passive avoidance test with the addition of naïve mice. Veh: vehicle. TFP: trifluoperazine.

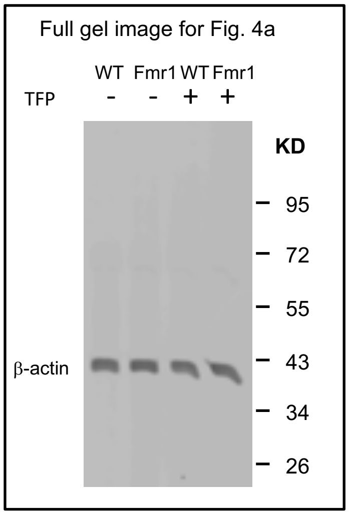
Full gel image for Fig. 4a
WT Fmr1 WT Fmr1
TFP - - + +
β-actin
KD
95
72
55
43
34
26

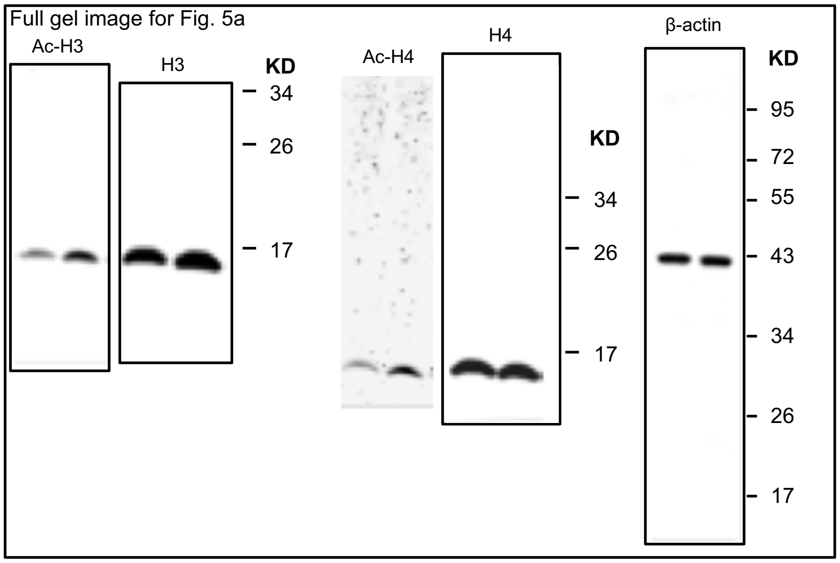
Full gel image for Fig. 5a
Ac-H3
H3
KD
34
26
17
Ac-H4
H4
KD
34
26
17
β-actin
KD
95
72
55
43
34
26
17

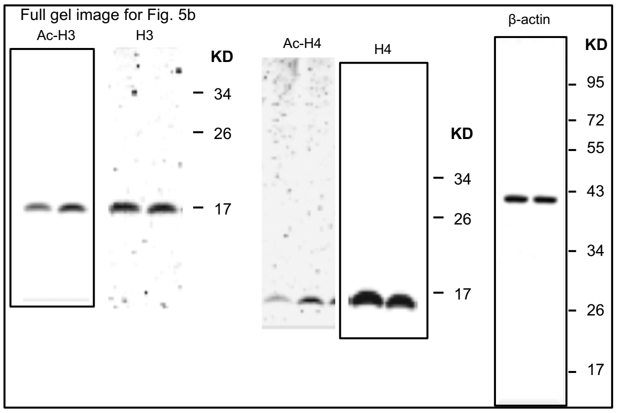
Full gel image for Fig. 5b
Ac-H3
H3
KD
34
26
17
Ac-H4
H4
KD
34
26
17
β-actin
KD
95
72
55
43
34
26
17

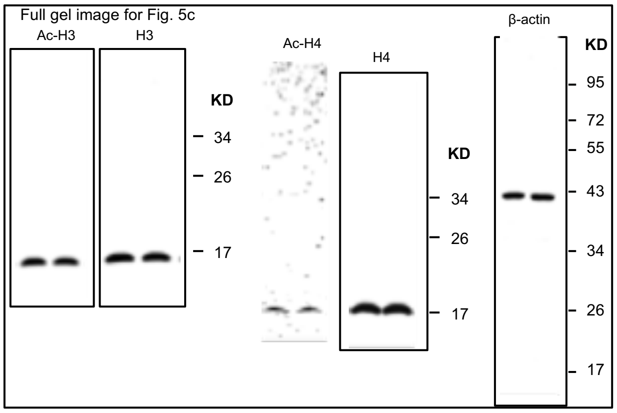
Full gel image for Fig. 5c
Ac-H3
H3
KD
34
26
17
Ac-H4
H4
KD
34
26
17
β-actin
KD
95
72
55
43
34
26
17

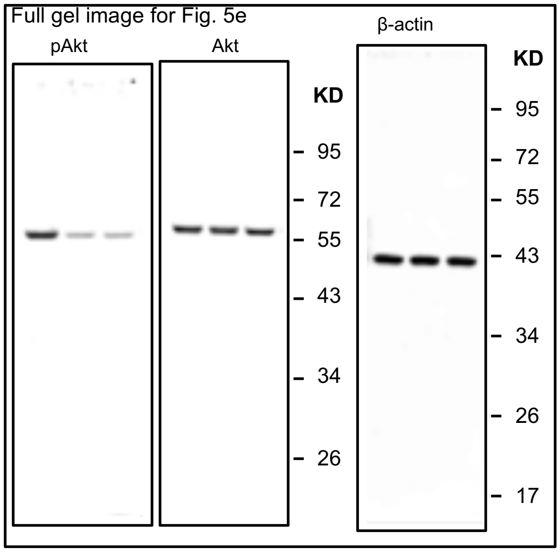
Full gel image for Fig. 5e
pAkt
Akt
KD
95
72
55
43
34
26
β-actin
KD
95
72
55
43
34
26
17

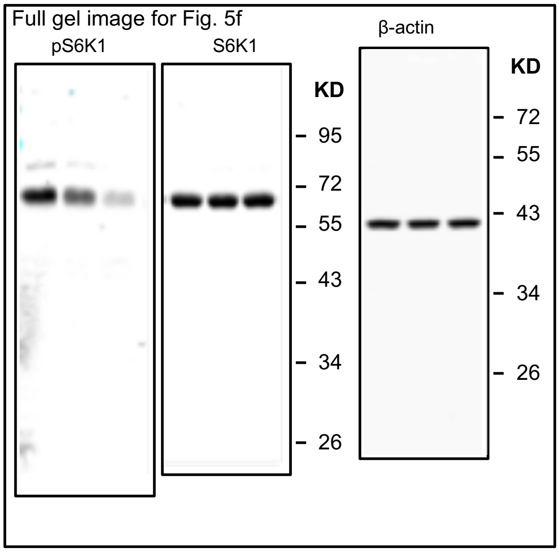
Full gel image for Fig. 5f
pS6K1
S6K1
KD
95
72
55
43
34
26
β-actin
KD
72
55
43
34
26

Full gel image for Fig. 6

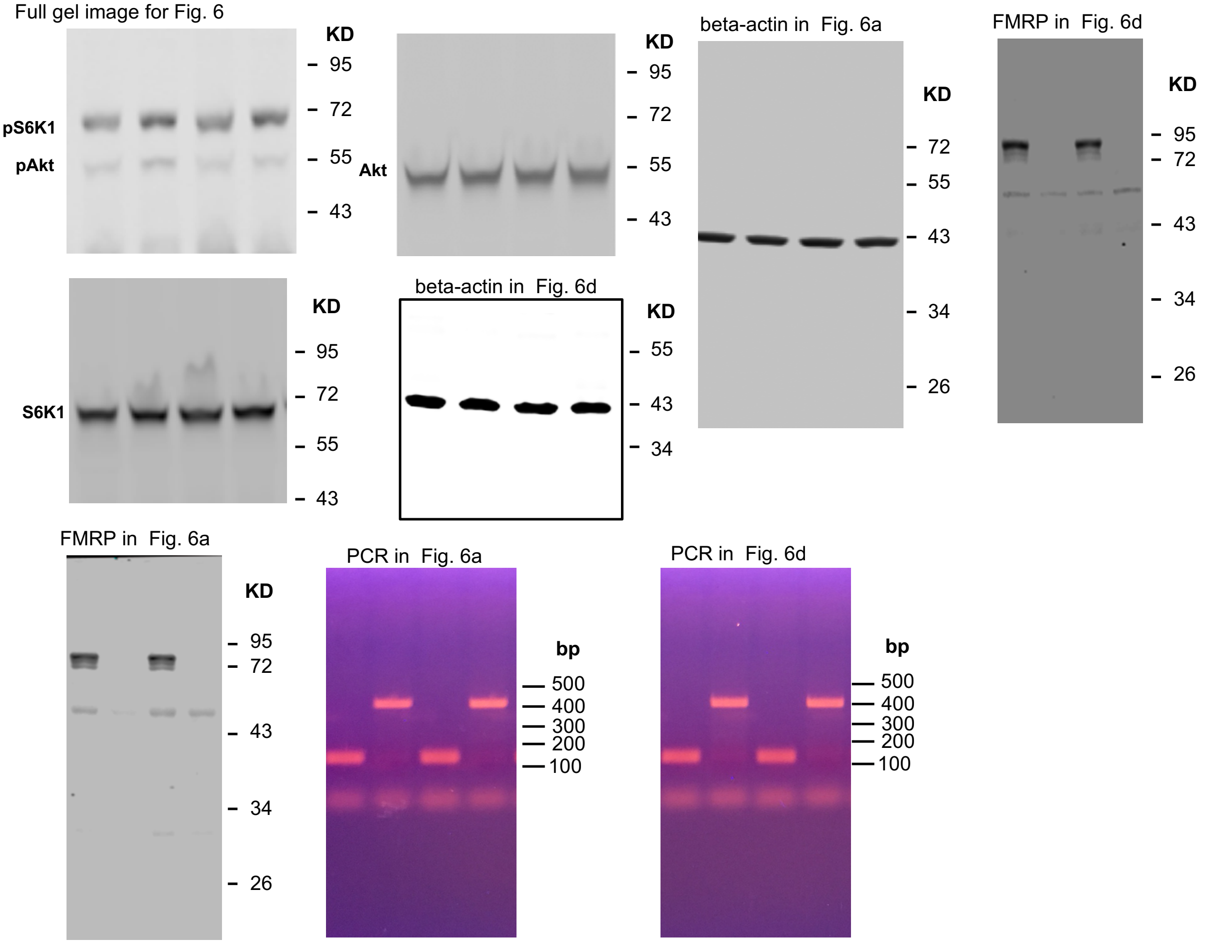

Full gel image for Fig. 7a

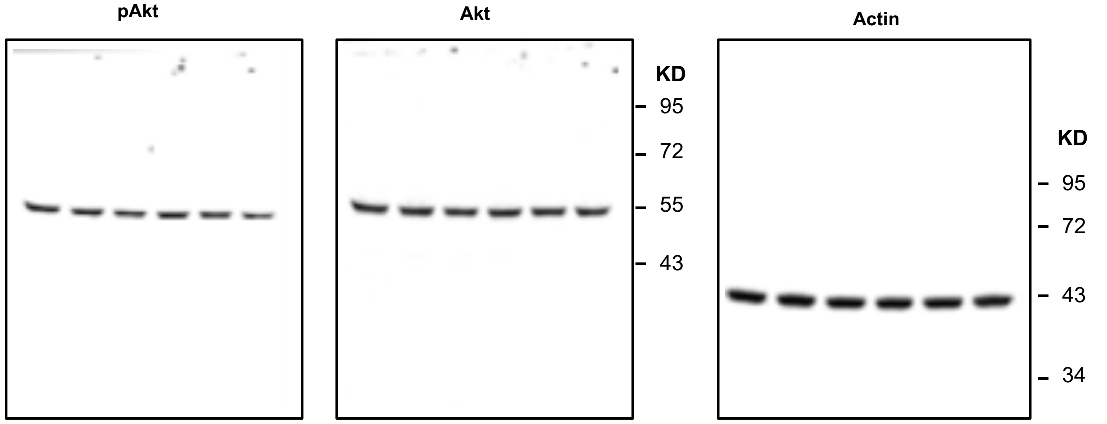

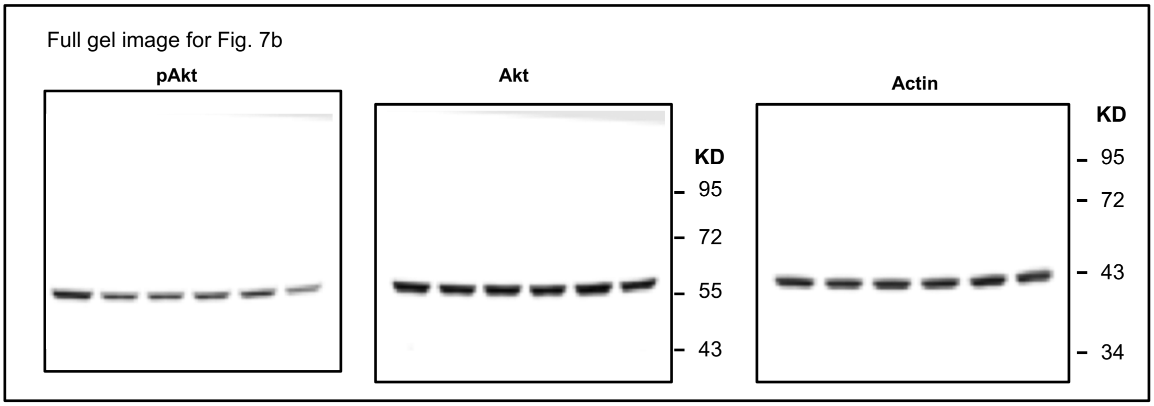

Supplementary Figure 13 Full Western blot images in Fig. 4, 5, 6, and 7 (as indicated) are shown.

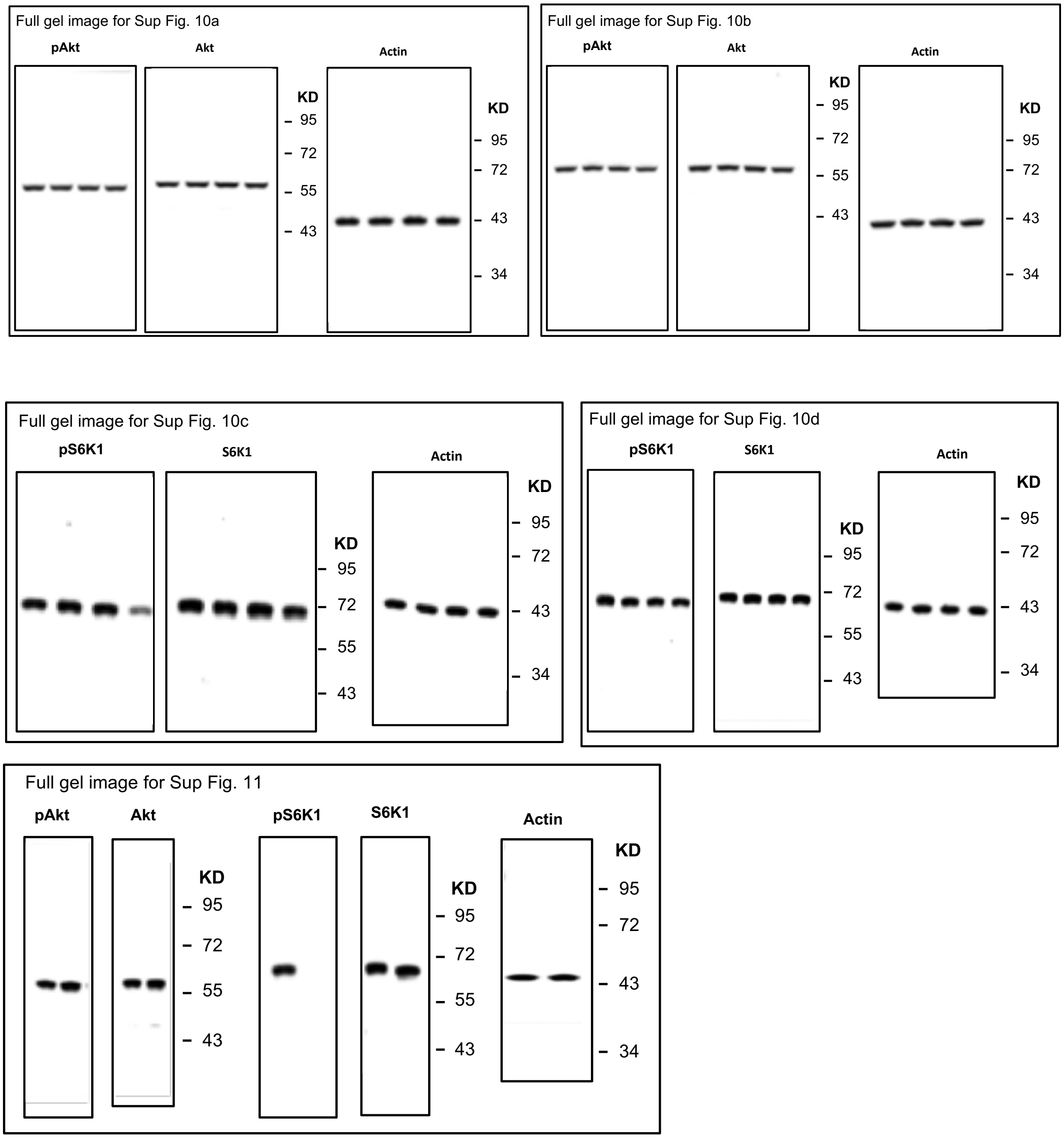

Supplementary Figure 14 Full Western blot images in Supplementary Fig. 10 and 11 (as indicated) are shown.
