# Supplementary material for: Transcriptome signature analysis repurposes trifluoperazine for the treatment of fragile X syndrome in mouse model
Source: Commun Biol. 2020 Mar 16;3:127. doi: 10.1038/s42003-020-0833-4 (PMC7075969; doi:10.1038/s42003-020-0833-4)
Supplement: Supplementary file 2 — Description of Additional Supplementary Files [file 42003_2020_833_MOESM2_ESM.pdf]

Description of Supplementary data

Supplementary Data 1 List of differentially expressed genes (DEGs) between wild type and Fmr1 knockout neurons. WT: samples from wild type hippocampal neuron. FMR: samples from *Fmr1* knockout (KO) hippocampal neuron. DEG list includes transcripts that are significantly up- or down-regulated in *Fmr1* KO samples ($P < 0.05$). Transcripts that show FPKM (Fragments Per Kilobase of transcript per Million mapped reads) less than 1 in both WT and FMR samples are excluded.

Supplementary Data 2 Complete list of GO biology processes associated with differentially expressed genes (DEG) in Fmr1 knockout hippocampal neurons. The list of GO categories is arranged in an ascending order of P values ($P<0.05$ is considered significant). Count represents the number of DEG detected in the specified GO process. % represents the percentage of total number of genes involved in the specified GO process.

Supplementary Data 3 Genes in the top 15 GO processes associated with DEGs in Fmr1 knockout (KO) hippocampal neurons. Changes of transcript level (ratio of *Fmr1* KO to wild type value) are expressed as Log2 values.

Supplementary Data 4 Genes in the KEGG pathways associated with differentially expressed genes (DEG) in *Fmr1* knockout (KO) hippocampal neurons. KEGG pathways are ranked and listed in an ascending order of P values ($P < 0.05$ is considered significant, as shown in Fig. 1C). Changes of transcript level (ratio of *Fmr1* KO to wild type value) are expressed as Log2 values.

Supplementary Data 5 Ranking of CMAP compounds, which trigger similar transcriptome alterations (including positive and negative correlations) to that in *Fmr1* knockout hippocampal neurons. Ranking is arranged in an ascending order of p-value, and then a descending order of (absolute) enrichment when two p-values are identical. Compound name and cell line indicate the name of compound used for

treatment with specific cell lines in CMAP database. The similarity mean is the arithmetic mean of the similarity/connectivity scores for all instances of the specific named compound. Number of arrays (N) is the number of analyzed arrays obtained from all instances of the corresponding compound/drug. Enrichment score is a measure of enrichment of DEG between *Fmr1* KO samples and all instances of the specific CMAP compound. It is computed using the Kolmogorov-Smirnov statistic as described 38. Compound with negative similarity mean triggers oppositional transcriptome changes comparing to the change caused by FMRP deficiency in Fmr1 knockout neurons. Compound with positive similarity mean triggers transcriptome changes overlapping with that caused by FMRP deficiency in *Fmr1* knockout neurons.

Supplementary Data 6 Summary of signature probes corresponding to up-regulated genes and down-regulated genes in PC3 cell treated with trifluoperazine.

Supplementary Data 7 Ranking of CMAP compounds, which trigger similar transcriptome alterations (including positive and negative correlations) to that triggered by trifluoperazine. Signature probes of trifluoperazine in PC3 cell are used as query. Compound with positive and negative similarity mean triggers overlapping and oppositional transcriptome changes, respectively, comparing to the change caused by trifluoperazine in PC3 cell. Rank, p value, enrichment score, and similarity mean are described in Supplementary Table 4. Compound name and cell line indicate the name of compound used for treatment with specific cell lines in CMAP database. Number of arrays (N) is the number of analyzed arrays obtained from all instances of the corresponding compound/drug.

Supplementary Data 8 Summary of signature probes corresponding to up-regulated genes and down-regulated genes in *Fmr1* knockout hippocampal neurons.

Supplementary Data 9 Data source for graphs in Figures 2 to 8.

Supplementary Data 10 Data source for graphs in Supplementary Figures 1 to 11.
