# Supplementary material for: Transcriptome signature analysis repurposes trifluoperazine for the treatment of fragile X syndrome in mouse model
Source: Commun Biol. 2020 Mar 16;3:127. doi: 10.1038/s42003-020-0833-4 (PMC7075969; doi:10.1038/s42003-020-0833-4)
Supplement: Supplementary file 4 — Supplementary Data 2 [file 42003_2020_833_MOESM4_ESM.pdf]

Supplementary Data 2

Rank	GO Biology Process	Count	%	P-Value
1	cell adhesion	54	4.2	1.70E-06
2	positive regulation of cytosolic calcium ion concentration	25	2	1.90E-06
3	nervous system development	41	3.2	6.10E-05
4	negative regulation of neuron apoptotic process	23	1.8	7.20E-05
5	positive regulation of cell migration	26	2	1.50E-04
6	cell cycle	57	4.5	1.60E-04
7	cell division	39	3.1	2.20E-04
8	cellular response to mechanical stimulus	14	1.1	3.20E-04
9	mitotic nuclear division	31	2.4	3.40E-04
10	regulation of G-protein coupled receptor protein signaling pathway	9	0.7	4.30E-04
11	positive regulation of GTPase activity	20	1.6	4.50E-04
12	positive chemotaxis	6	0.5	9.70E-04
13	positive regulation of endothelial cell proliferation	12	0.9	1.00E-03
14	regulation of ion transmembrane transport	18	1.4	1.30E-03
15	response to estradiol	15	1.2	1.30E-03
16	positive regulation of synaptic transmission, GABAergic	6	0.5	1.30E-03
17	wound healing	14	1.1	2.00E-03
18	response to mechanical stimulus	11	0.9	2.30E-03
19	odontogenesis of dentin-containing tooth	11	0.9	2.30E-03
20	single organismal cell-cell adhesion	15	1.2	2.50E-03
21	ERK1 and ERK2 cascade	7	0.5	2.50E-03
22	cell differentiation	63	4.9	2.50E-03
23	response to oxidative stress	17	1.3	2.80E-03
24	hepatocyte proliferation	4	0.3	3.00E-03
25	regulation of axon diameter	4	0.3	3.00E-03
26	sequestering of actin monomers	5	0.4	3.20E-03
27	positive regulation of actin filament bundle assembly	5	0.4	3.20E-03
28	axon guidance	18	1.4	3.60E-03
29	potassium ion transmembrane transport	13	1	3.90E-03
30	positive regulation of neuron projection development	17	1.3	4.00E-03
31	oxidation-reduction process	55	4.3	4.20E-03
32	positive regulation of vasodilation	8	0.6	4.30E-03
33	potassium ion transport	16	1.3	4.40E-03
34	xenophagy	14	1.1	4.40E-03
35	mitotic chromosome condensation	5	0.4	4.40E-03
36	positive regulation of cAMP biosynthetic process	9	0.7	5.10E-03
37	kidney development	16	1.3	5.50E-03
38	negative regulation of cell migration	14	1.1	5.60E-03

39	receptor internalization	8	0.6	5.80E-03
40	response to cold	8	0.6	5.80E-03
41	cytoskeleton-dependent intracellular transport	5	0.4	6.00E-03
42	negative regulation of protein kinase activity	13	1	6.10E-03
43	mitophagy in response to mitochondrial depolarization	16	1.3	6.30E-03
44	positive regulation of apoptotic process	31	2.4	6.50E-03
45	positive regulation of protein phosphorylation	20	1.6	6.60E-03
46	fatty acid biosynthetic process	11	0.9	7.40E-03
47	neuron projection morphogenesis	10	0.8	7.50E-03
48	positive regulation of penile erection	4	0.3	7.70E-03
49	astrocyte cell migration	4	0.3	7.70E-03
50	response to estrogen	11	0.9	8.10E-03
51	negative regulation of cell proliferation	34	2.7	8.20E-03
52	negative regulation of signal transduction	9	0.7	8.20E-03
53	cellular response to organic substance	7	0.5	8.60E-03
54	brain development	22	1.7	8.70E-03
55	negative regulation of MAP kinase activity	8	0.6	8.70E-03
56	positive regulation of synaptic transmission, glutamatergic	6	0.5	9.00E-03
57	lung morphogenesis	6	0.5	9.00E-03
58	chromosome segregation	12	0.9	9.90E-03
59	response to amphetamine	7	0.5	1.00E-02
60	extracellular matrix organization	14	1.1	1.00E-02
61	apoptotic process	46	3.6	1.00E-02
62	positive regulation of vasculogenesis	4	0.3	1.10E-02
63	positive regulation of adenylate cyclase activity involved in G-protein coupled receptor signaling pathway	4	0.3	1.10E-02
64	negative regulation of Wnt signaling pathway	9	0.7	1.10E-02
65	positive regulation of MAPK cascade	13	1	1.20E-02
66	positive regulation of osteoblast differentiation	10	0.8	1.20E-02
67	synaptic vesicle endocytosis	5	0.4	1.20E-02
68	negative regulation of cell death	11	0.9	1.30E-02
69	branching morphogenesis of an epithelial tube	7	0.5	1.30E-02
70	negative regulation of cell cycle	7	0.5	1.30E-02
71	angiogenesis	23	1.8	1.30E-02
72	lipid metabolic process	38	3	1.40E-02
73	positive regulation of synapse assembly	10	0.8	1.50E-02
74	lipid storage	6	0.5	1.50E-02
75	skeletal system development	13	1	1.50E-02
76	mitotic spindle assembly checkpoint	5	0.4	1.50E-02
77	aging	18	1.4	1.50E-02
78	cellular response to corticotropin-releasing hormone stimulus	3	0.2	1.70E-02

79	negative regulation of hydrogen peroxide-mediated programmed cell death	3	0.2	1.70E-02
80	adherens junction maintenance	3	0.2	1.70E-02
81	multicellular organism development	74	5.8	1.70E-02
82	regulation of heart rate by cardiac conduction	6	0.5	1.70E-02
83	positive regulation of defense response to virus by host	14	1.1	1.70E-02
84	behavioral response to cocaine	5	0.4	1.90E-02
85	cell migration	19	1.5	1.90E-02
86	renal system process	4	0.3	2.00E-02
87	labyrinthine layer development	4	0.3	2.00E-02
88	protein localization to plasma membrane	9	0.7	2.00E-02
89	substrate adhesion-dependent cell spreading	7	0.5	2.20E-02
90	response to progesterone	6	0.5	2.30E-02
91	negative regulation of neuron projection development	9	0.7	2.40E-02
92	calcium ion transport	15	1.2	2.40E-02
93	embryonic skeletal joint morphogenesis	4	0.3	2.50E-02
94	actin crosslink formation	4	0.3	2.50E-02
95	regulation of G2/M transition of mitotic cell cycle	4	0.3	2.50E-02
96	regulation of cell migration	10	0.8	2.60E-02
97	negative regulation of GTPase activity	6	0.5	2.60E-02
98	positive regulation of fibroblast proliferation	9	0.7	2.60E-02
99	amino acid transport	7	0.5	2.70E-02
100	regulation of synaptic plasticity	7	0.5	2.70E-02
101	behavioral fear response	7	0.5	2.70E-02
102	epithelial cell differentiation	9	0.7	3.10E-02
103	morphogenesis of embryonic epithelium	4	0.3	3.20E-02
104	inhibitory postsynaptic potential	4	0.3	3.20E-02
105	negative regulation of cardiac muscle cell proliferation	4	0.3	3.20E-02
106	negative regulation of G-protein coupled receptor protein signaling pathway	4	0.3	3.20E-02
107	embryonic skeletal system development	7	0.5	3.30E-02
108	positive regulation of cytokinesis	6	0.5	3.40E-02
109	cellular response to estradiol stimulus	6	0.5	3.40E-02
110	response to cytokine	10	0.8	3.40E-02
111	female pregnancy	10	0.8	3.40E-02
112	positive regulation of angiogenesis	13	1	3.60E-02
113	positive regulation of vasoconstriction	7	0.5	3.60E-02
114	ovarian follicle development	8	0.6	3.70E-02
115	semaphorin-plexin signaling pathway	6	0.5	3.80E-02
116	positive regulation of neurogenesis	6	0.5	3.80E-02
117	endothelial cell morphogenesis	4	0.3	3.90E-02
118	tissue remodeling	4	0.3	3.90E-02
119	negative regulation of potassium ion transport	4	0.3	3.90E-02

120	cell activation	4	0.3	3.90E-02
121	cerebral cortex GABAergic interneuron migration	3	0.2	3.90E-02
122	intermediate filament bundle assembly	3	0.2	3.90E-02
123	Toll signaling pathway	3	0.2	3.90E-02
124	astrocyte activation	3	0.2	3.90E-02
125	regulation of cell growth	8	0.6	4.00E-02
126	positive regulation of transcription, DNA-templated	43	3.4	4.10E-02
127	negative regulation of microtubule depolymerization	5	0.4	4.10E-02
128	DNA replication initiation	5	0.4	4.10E-02
129	smoothened signaling pathway	9	0.7	4.20E-02
130	cellular response to BMP stimulus	6	0.5	4.20E-02
131	cellular response to DNA damage stimulus	33	2.6	4.20E-02
132	sensory perception of sound	14	1.1	4.20E-02
133	neuronal stem cell population maintenance	5	0.4	4.70E-02
134	negative regulation of peptidyl-serine phosphorylation	5	0.4	4.70E-02
135	regulation of double-strand break repair via homologous recombination	4	0.3	4.70E-02
136	osteoblast differentiation	12	0.9	4.80E-02
137	ion transport	43	3.4	4.90E-02
138	neuron differentiation	13	1	4.90E-02
