# Supplementary material for: Transcriptome signature analysis repurposes trifluoperazine for the treatment of fragile X syndrome in mouse model
Source: Commun Biol. 2020 Mar 16;3:127. doi: 10.1038/s42003-020-0833-4 (PMC7075969; doi:10.1038/s42003-020-0833-4)
Supplement: Supplementary file 6 — Supplementary Data 4 [file 42003_2020_833_MOESM6_ESM.pdf]

Supplementary Data 4

Cell cycle		
**Symbol**	**Gene name**	**Log2 (fold change)**
Bub1	BUB1, mitotic checkpoint serine/threonine kinase(Bub1)	0.446547
Bub1b	BUB1B, mitotic checkpoint serine/threonine kinase(Bub1b)	0.230577
Cdc14a	CDC14 cell division cycle 14A(Cdc14a)	0.264674
Dbf4	DBF4 zinc finger(Dbf4)	0.281871
Mad2l1	MAD2 mitotic arrest deficient-like 1(Mad2l1)	0.23892
Cdc20	cell division cycle 20(Cdc20)	0.353063
Cdc6	cell division cycle 6(Cdc6)	0.476019
Chek1	checkpoint kinase 1(Chek1)	0.258348
Ccna2	cyclin A2(Ccna2)	0.261407
Ccnb2	cyclin B2(Ccnb2)	0.257691
Ccnd1	cyclin D1(Ccnd1)	0.18533
Ccnd2	cyclin D2(Ccnd2)	0.353105
Ccne1	cyclin E1(Ccne1)	-0.260069
Cdkn1c	cyclin-dependent kinase inhibitor 1C (P57)(Cdkn1c)	-0.539388
Gadd45g	growth arrest and DNA-damage-inducible 45 gamma(Gadd45g)	-0.254925
Mcm2	minichromosome maintenance complex component 2(Mcm2)	0.233332
Mcm5	minichromosome maintenance complex component 5(Mcm5)	0.271372
Mcm7	minichromosome maintenance complex component 7(Mcm7)	0.316533
Pttg1	pituitary tumor-transforming gene 1(Pttg1)	-0.268946
Plk1	polo-like kinase 1(Plk1)	0.270134
**MAPK signaling pathway**		
**Symbol**	**Gene name**	**Log2 (fold change)**
Elk1	ELK1, member of ETS oncogene family(Elk1)	0.190294
Rasgrp1	RAS guanyl releasing protein 1(Rasgrp1)	-0.302872
Rapgef2	Rap guanine nucleotide exchange factor (GEF) 2(Rapgef2)	0.18849
Arrb1	arrestin, beta 1(Arrb1)	0.191243
Relb	avian reticuloendotheliosis viral (v-rel) oncogene related B(Relb)	-0.481266
Bdnf	brain derived neurotrophic factor(Bdnf)	-0.470373
Cacna1i	calcium channel, voltage-dependent, alpha 1I subunit(Cacna1i)	-0.194993
Cacng3	calcium channel, voltage-dependent, gamma subunit 3(Cacng3)	-0.419447

Cacng4	calcium channel, voltage-dependent, gamma subunit 4(Cacng4)	0.234454
Cacng5	calcium channel, voltage-dependent, gamma subunit 5(Cacng5)	-0.44742
Dusp4	dual specificity phosphatase 4(Dusp4)	-0.315051
Dusp7	dual specificity phosphatase 7(Dusp7)	-0.208387
Egfr	epidermal growth factor receptor(Egfr)	0.283762
Fgf9	fibroblast growth factor 9(Fgf9)	-0.394831
Flna	filamin, alpha(Flna)	0.203053
Gadd45g	growth arrest and DNA-damage-inducible 45 gamma(Gadd45g)	-0.254925
Hspa2	heat shock protein 2(Hspa2)	-0.320115
Mapk12	mitogen-activated protein kinase 12(Mapk12)	-0.587148
Map2k1	mitogen-activated protein kinase kinase 1(Map2k1)	-0.23032
Map3k1	mitogen-activated protein kinase kinase kinase 1(Map3k1)	0.19665
Map4k4	mitogen-activated protein kinase kinase kinase kinase 4(Map4k4)	0.193833
Nr4a1	nuclear receptor subfamily 4, group A, member 1(Nr4a1)	-0.315837
Pdgfra	platelet derived growth factor receptor, alpha polypeptide(Pdgfra)	0.288914
Rras	related RAS viral (r-ras) oncogene(Rras)	-0.397615
Rps6ka5	ribosomal protein S6 kinase, polypeptide 5(Rps6ka5)	0.239409
Stmn1	stathmin 1(Stmn1)	0.194703
Tgfbr2	transforming growth factor, beta receptor II(Tgfbr2)	-0.402446
**Focal adhesion**		
**Symbol**	**Gene name**	**Log2 (fold change)**
Elk1	ELK1, member of ETS oncogene family(Elk1)	0.190294
Cav1	caveolin 1, caveolae protein(Cav1)	-0.33376
Col2a1	collagen, type II, alpha 1(Col2a1)	-0.495529
Col5a1	collagen, type V, alpha 1(Col5a1)	0.188501
Col6a1	collagen, type VI, alpha 1(Col6a1)	-0.314202
Col6a2	collagen, type VI, alpha 2(Col6a2)	-0.668208
Ccnd1	cyclin D1(Ccnd1)	0.18533
Ccnd2	cyclin D2(Ccnd2)	0.353105
Egfr	epidermal growth factor receptor(Egfr)	0.283762
Fn1	fibronectin 1(Fn1)	0.312679
Flna	filamin, alpha(Flna)	0.203053
Hgf	hepatocyte growth factor(Hgf)	0.423115
Itgav	integrin alpha V(Itgav)	0.229906
Lama5	laminin, alpha 5(Lama5)	0.20888
Lamc2	laminin, gamma 2(Lamc2)	-0.526442
Met	met proto-oncogene(Met)	0.198161

Map2k1	mitogen-activated protein kinase kinase 1(Map2k1)	-0.23032
Mylk	myosin, light polypeptide kinase(Mylk)	-0.397011
Parvb	parvin, beta(Parvb)	-0.268398
Pdgfra	platelet derived growth factor receptor, alpha polypeptide(Pdgfra)	0.288914
Pdgfd	platelet-derived growth factor, D polypeptide(Pdgfd)	-0.561035
Thbs3	thrombospondin 3(Thbs3)	0.384423
Zyx	zyxin(Zyx)	-0.210285
**p53 signaling pathway**		
**Symbol**	**Gene name**	**Log2 (fold change)**
Cd82	CD82 antigen(Cd82)	-0.584949
Gtse1	G two S phase expressed protein 1(Gtse1)	0.388941
Chek1	checkpoint kinase 1(Chek1)	0.258348
Ccnb2	cyclin B2(Ccnb2)	0.257691
Ccnd1	cyclin D1(Ccnd1)	0.18533
Ccnd2	cyclin D2(Ccnd2)	0.353105
Ccne1	cyclin E1(Ccne1)	-0.260069
Gadd45g	growth arrest and DNA-damage-inducible 45 gamma(Gadd45g)	-0.254925
Pidd1	p53 induced death domain protein 1(Pidd1)	0.46691
Rrm2	ribonucleotide reductase M2(Rrm2)	0.23946
Shisa5	shisa family member 5(Shisa5)	-0.260195
**Calcium signaling pathway**		
**Symbol**	**Gene name**	**Log2 (fold change)**
Htr6	5-hydroxytryptamine (serotonin) receptor 6(Htr6)	0.400014
Atp2b4	ATPase, Ca++ transporting, plasma membrane 4(Atp2b4)	-0.209647
Adora2a	adenosine A2a receptor(Adora2a)	-0.284827
Adcy8	adenylate cyclase 8(Adcy8)	-0.219063
Adra1b	adrenergic receptor, alpha 1b(Adra1b)	-0.34772
Cacna1i	calcium channel, voltage-dependent, alpha 1I subunit(Cacna1i)	-0.194993
Cckbr	cholecystokinin B receptor(Cckbr)	-0.562344
F2r	coagulation factor II (thrombin) receptor(F2r)	-0.221243
Egfr	epidermal growth factor receptor(Egfr)	0.283762
Itpka	inositol 1,4,5-trisphosphate 3-kinase A(Itpka)	-0.304801
Itpkb	inositol 1,4,5-trisphosphate 3-kinase B(Itpkb)	0.200464
Mylk	myosin, light polypeptide kinase(Mylk)	-0.397011
Nos1	nitric oxide synthase 1, neuronal(Nos1)	0.450857
Oxtr	oxytocin receptor(Oxtr)	0.267845
Plcd3	phospholipase C, delta 3(Plcd3)	-0.264772
Pdgfra	platelet derived growth factor receptor, alpha	0.288914

	polypeptide(Pdgfra)	
P2rx3	purinergic receptor P2X, ligand-gated ion channel, 3(P2rx3)	-0.297367
P2rx6	purinergic receptor P2X, ligand-gated ion channel, 6(P2rx6)	-0.302309
Tacr3	tachykinin receptor 3(Tacr3)	-0.345044
Trhr	thyrotropin releasing hormone receptor(Trhr)	-0.253903
**Amino sugar and nucleotide sugar metabolism**		
**Symbol**	**Gene name**	**Log2 (fold change)**
Gmds	GDP-mannose 4, 6-dehydratase(Gmds)	-0.273588
Nans	N-acetylneuraminic acid synthase (sialic acid synthase)(Nans)	-0.28157
Cyb5r1	cytochrome b5 reductase 1(Cyb5r1)	-0.333184
Cyb5r2	cytochrome b5 reductase 2(Cyb5r2)	-0.686573
Fpgt	fucose-1-phosphate guanylyltransferase(Fpgt)	-0.231752
Gck	glucokinase(Gck)	-0.33515
Hexb	hexosaminidase B(Hexb)	-0.446539
Renbp	renin binding protein(Renbp)	-0.62755
**Cocaine addiction**		
**Symbol**	**Gene name**	**Log2 (fold change)**
Gpsm1	G-protein signalling modulator 1 (AGS3-like, C. elegans)(Gpsm1)	0.267821
Adcy5	adenylate cyclase 5(Adcy5)	0.18128
Bdnf	brain derived neurotrophic factor(Bdnf)	-0.470373
Grm2	glutamate receptor, metabotropic 2(Grm2)	-0.408961
Maob	monoamine oxidase B(Maob)	-0.607282
Pdyn	prodynorphin(Pdyn)	-0.715908
Ppp1r1b	protein phosphatase 1, regulatory (inhibitor) subunit 1B(Ppp1r1b)	0.269958
Slc18a2	solute carrier family 18 (vesicular monoamine), member 2(Slc18a2)	-0.364674
**Rap1 signaling pathway**		
**Symbol**	**Gene name**	**Log2 (fold change)**
Rapgef2	Rap guanine nucleotide exchange factor (GEF) 2(Rapgef2)	0.18849
Rapgef3	Rap guanine nucleotide exchange factor (GEF) 3(Rapgef3)	0.305563
Adora2a	adenosine A2a receptor(Adora2a)	-0.284827
Adcy5	adenylate cyclase 5(Adcy5)	0.18128
Adcy8	adenylate cyclase 8(Adcy8)	-0.219063
Afdn	afadin, adherens junction formation factor(Afdn)	0.202055
F2r	coagulation factor II (thrombin) receptor(F2r)	-0.221243
Dock4	dedicator of cytokinesis 4(Dock4)	0.203538
Egfr	epidermal growth factor receptor(Egfr)	0.283762
Fgf9	fibroblast growth factor 9(Fgf9)	-0.394831

Hgf	hepatocyte growth factor(Hgf)	0.423115
Id1	inhibitor of DNA binding 1(Id1)	-0.589363
Lpar4	lysophosphatidic acid receptor 4(Lpar4)	0.46842
Met	met proto-oncogene(Met)	0.198161
Mapk12	mitogen-activated protein kinase 12(Mapk12)	-0.587148
Map2k1	mitogen-activated protein kinase kinase 1(Map2k1)	-0.23032
Pdgfra	platelet derived growth factor receptor, alpha polypeptide(Pdgfra)	0.288914
Pdgfd	platelet-derived growth factor, D polypeptide(Pdgfd)	-0.561035
P2ry1	purinergic receptor P2Y, G-protein coupled 1(P2ry1)	-0.317979
Rras	related RAS viral (r-ras) oncogene(Rras)	-0.397615
**HTLV-I infection**		
**Symbol**	**Gene name**	**Log2 (fold change)**
Bub1b	BUB1B, mitotic checkpoint serine/threonine kinase(Bub1b)	0.230577
Crtc2	CREB regulated transcription coactivator 2(Crtc2)	0.228122
Ets1	E26 avian leukemia oncogene 1, 5' domain(Ets1)	0.253717
Elk1	ELK1, member of ETS oncogene family(Elk1)	0.190294
Mad2l1	MAD2 mitotic arrest deficient-like 1(Mad2l1)	0.23892
Apc2	adenomatosis polyposis coli 2(Apc2)	0.236055
Apc	adenomatosis polyposis coli(Apc)	0.311095
Adcy5	adenylate cyclase 5(Adcy5)	0.18128
Adcy8	adenylate cyclase 8(Adcy8)	-0.219063
Relb	avian reticuloendotheliosis viral (v-rel) oncogene related B(Relb)	-0.481266
Cdc20	cell division cycle 20(Cdc20)	0.353063
Chek1	checkpoint kinase 1(Chek1)	0.258348
Ccnd1	cyclin D1(Ccnd1)	0.18533
Ccnd2	cyclin D2(Ccnd2)	0.353105
Dvl1	dishevelled segment polarity protein 1(Dvl1)	0.183879
H2-T24	histocompatibility 2, T region locus 24(H2-T24)	-0.347115
Map3k1	mitogen-activated protein kinase kinase kinase 1(Map3k1)	0.19665
Mybl2	myeloblastosis oncogene-like 2(Mybl2)	0.389053
Pttg1	pituitary tumor-transforming gene 1(Pttg1)	-0.268946
Pdgfra	platelet derived growth factor receptor, alpha polypeptide(Pdgfra)	0.288914
Pole	polymerase (DNA directed), epsilon(Pole)	0.2455
Rras	related RAS viral (r-ras) oncogene(Rras)	-0.397615
Trp53inp1	transformation related protein 53 inducible nuclear protein 1(Trp53inp1)	0.269027
Tgfbr2	transforming growth factor, beta receptor II(Tgfbr2)	-0.402446
**Neuroactive ligand-receptor interaction**		
**Symbol**	**Gene name**	**Log2 (fold**

		change)
Htr6	5-hydroxytryptamine (serotonin) receptor 6(Htr6)	0.400014
Adora2a	adenosine A2a receptor(Adora2a)	-0.284827
Adra1b	adrenergic receptor, alpha 1b(Adra1b)	-0.34772
Adra2a	adrenergic receptor, alpha 2a(Adra2a)	0.205162
Cckbr	cholecystokinin B receptor(Cckbr)	-0.562344
F2r	coagulation factor II (thrombin) receptor(F2r)	-0.221243
Crhr1	corticotropin releasing hormone receptor 1(Crhr1)	-0.311301
Gabra2	gamma-aminobutyric acid (GABA) A receptor, subunit alpha 2(Gabra2)	-1.9779
Gabrd	gamma-aminobutyric acid (GABA) A receptor, subunit delta(Gabrd)	-0.501806
Grik1	glutamate receptor, ionotropic, kainate 1(Grik1)	0.285063
Grm2	glutamate receptor, metabotropic 2(Grm2)	-0.408961
Grm8	glutamate receptor, metabotropic 8(Grm8)	-0.504899
Lpar4	lysophosphatidic acid receptor 4(Lpar4)	0.46842
Npy2r	neuropeptide Y receptor Y2(Npy2r)	-0.230785
Oxtr	oxytocin receptor(Oxtr)	0.267845
Pth1r	parathyroid hormone 1 receptor(Pth1r)	-0.735892
P2rx3	purinergic receptor P2X, ligand-gated ion channel, 3(P2rx3)	-0.297367
P2rx6	purinergic receptor P2X, ligand-gated ion channel, 6(P2rx6)	-0.302309
P2ry1	purinergic receptor P2Y, G-protein coupled 1(P2ry1)	-0.317979
P2ry2	purinergic receptor P2Y, G-protein coupled 2(P2ry2)	-0.415118
Sstr3	somatostatin receptor 3(Sstr3)	-0.225749
Tacr3	tachykinin receptor 3(Tacr3)	-0.345044
Thrb	thyroid hormone receptor beta(Thrb)	-0.223796
Trhr	thyrotropin releasing hormone receptor(Trhr)	-0.253903
**Pathways in cancer**		
**Symbol**	**Gene name**	**Log2 (fold change)**
Rasgrp1	RAS guanyl releasing protein 1(Rasgrp1)	-0.302872
Arhgef1	Rho guanine nucleotide exchange factor (GEF) 1(Arhgef1)	0.236485
Traf3	TNF receptor-associated factor 3(Traf3)	0.205892
Apc2	adenomatosis polyposis coli 2(Apc2)	0.236055
Apc	adenomatosis polyposis coli(Apc)	0.311095
Adcy5	adenylate cyclase 5(Adcy5)	0.18128
Adcy8	adenylate cyclase 8(Adcy8)	-0.219063
Birc5	baculoviral IAP repeat-containing 5(Birc5)	0.315857
Bmp4	bone morphogenetic protein 4(Bmp4)	-0.338676
F2r	coagulation factor II (thrombin) receptor(F2r)	-0.221243
Csf2ra	colony stimulating factor 2 receptor, alpha, low-affinity (granulocyte-macrophage)(Csf2ra)	0.294034
Ccnd1	cyclin D1(Ccnd1)	0.18533

Ccne1	cyclin E1(Ccne1)	-0.260069
Dvl1	dishevelled segment polarity protein 1(Dvl1)	0.183879
Egfr	epidermal growth factor receptor(Egfr)	0.283762
Fgf9	fibroblast growth factor 9(Fgf9)	-0.394831
Fn1	fibronectin 1(Fn1)	0.312679
Gng11	guanine nucleotide binding protein (G protein), gamma 11(Gng11)	-0.799539
Hgf	hepatocyte growth factor(Hgf)	0.423115
Itgav	integrin alpha V(Itgav)	0.229906
Lama5	laminin, alpha 5(Lama5)	0.20888
Lamc2	laminin, gamma 2(Lamc2)	-0.526442
Lpar4	lysophosphatidic acid receptor 4(Lpar4)	0.46842
Met	met proto-oncogene(Met)	0.198161
Map2k1	mitogen-activated protein kinase kinase 1(Map2k1)	-0.23032
Msh2	mutS homolog 2(Msh2)	-0.213602
Pdgfra	platelet derived growth factor receptor, alpha polypeptide(Pdgfra)	0.288914
Ptgs2	prostaglandin-endoperoxide synthase 2(Ptgs2)	-0.380668
Ret	ret proto-oncogene(Ret)	-0.284101
Runx1t1	runt-related transcription factor 1; translocated to, 1 (cyclin D-related)(Runx1t1)	0.218432
Tgfbr2	transforming growth factor, beta receptor II(Tgfbr2)	-0.402446
