# Supplementary material for: Transcriptome signature analysis repurposes trifluoperazine for the treatment of fragile X syndrome in mouse model
Source: Commun Biol. 2020 Mar 16;3:127. doi: 10.1038/s42003-020-0833-4 (PMC7075969; doi:10.1038/s42003-020-0833-4)
Supplement: Supplementary file 7 — Supplementary Data 5 [file 42003_2020_833_MOESM7_ESM.pdf]

Supplementary Data 5

Rank	Compound name and cell line	Similarity mean	N	Enrichment	P
1	trichostatin A - PC3	-0.542	55	-0.661	0
2	tanespimycin - PC3	-0.486	12	-0.626	0
3	0175029-0000 - PC3	-0.725	4	-0.931	0.00002
4	methotrexate - MCF7	-0.828	3	-0.972	0.00008
5	etoposide - MCF7	-0.869	2	-0.993	0.00022
6	phenoxybenzamine - MCF7	-0.656	3	-0.947	0.00026
7	trifluoperazine - PC3	-0.741	3	-0.917	0.00096
8	luteolin - MCF7	-0.817	2	-0.975	0.00137
9	thioridazine - PC3	-0.635	5	-0.738	0.00248
10	Prestwick-642 - MCF7	0.608	2	0.958	0.00308
11	doxorubicin - MCF7	-0.744	2	-0.959	0.00364
12	mefloquine - PC3	-0.768	2	-0.958	0.0038
13	piribedil - MCF7	0.68	2	0.954	0.00392
14	chrysin - MCF7	-0.731	2	-0.957	0.00394
15	5230742 - MCF7	0.788	2	0.953	0.00404
16	pilocarpine - MCF7	0.617	2	0.951	0.00447
17	sulfafurazole - MCF7	0.312	3	0.865	0.00461
18	0316684-0000 - MCF7	0.625	2	0.948	0.00495
19	15-delta prostaglandin J2 - PC3	-0.641	3	-0.864	0.00511
20	GW-8510 - PC3	-0.748	2	-0.947	0.00614
21	betonicine - MCF7	0.41	3	0.849	0.00645
22	thiamine - MCF7	0.586	2	0.94	0.0068
23	lobelanidine - MCF7	0.597	2	0.938	0.00742
24	cloperastine - MCF7	-0.614	3	-0.841	0.00809
25	PF-00562151-00 - PC3	-0.43	4	-0.737	0.00949
26	cefazolin - PC3	-0.621	2	-0.93	0.01014
27	aztreonam - MCF7	-0.665	2	-0.927	0.01097
28	mycophenolic acid - MCF7	-0.656	2	-0.927	0.01109
29	alsterpaullone - PC3	-0.75	2	-0.925	0.01159
30	thiostrepton - MCF7	-0.612	2	-0.924	0.01181
31	PHA-00767505E - MCF7	0.543	2	0.92	0.01264
32	astemizole - PC3	-0.795	2	-0.921	0.01266
33	0173570-0000 - PC3	-0.592	4	-0.715	0.01337
34	nordihydroguaiaretic acid - MCF7	-0.449	8	-0.526	0.0137
35	troglitazone - PC3	-0.434	4	-0.713	0.01383
36	NU-1025 - MCF7	0.537	2	0.916	0.01404
37	fenbufen - PC3	-0.638	2	-0.912	0.01555
38	geldanamycin - MCF7	-0.246	10	-0.467	0.01617
39	perphenazine - PC3	-0.601	2	-0.909	0.0167
40	amitriptyline - MCF7	-0.623	3	-0.796	0.01723
41	tridihexethyl - MCF7	0.542	2	0.908	0.01748
42	moroxydine - MCF7	0.549	2	0.907	0.01785
43	amylocaine - MCF7	0.529	2	0.907	0.01801
44	chlorcyclizine - MCF7	-0.506	3	-0.792	0.01823
45	triamcinolone - MCF7	-0.607	2	-0.904	0.01845

46	glafenine - MCF7	0.494	2	0.905	0.01847
47	levomepromazine - PC3	-0.653	2	-0.903	0.01869
48	resveratrol - MCF7	-0.509	6	-0.582	0.01873
49	0175029-0000 - MCF7	-0.687	2	-0.902	0.01899
50	etofylline - PC3	0.669	2	0.903	0.01905
51	monorden - PC3	-0.466	5	-0.625	0.01933
52	ivermectin - MCF7	-0.598	2	-0.901	0.01968
53	propofol - MCF7	-0.613	2	-0.901	0.01976
54	thiethylperazine - MCF7	-0.596	2	-0.899	0.0203
55	benfluorex - MCF7	0.598	2	0.9	0.0204
56	alvespimycin - PC3	-0.658	2	-0.898	0.02066
57	dinoprost - MCF7	0.544	2	0.898	0.02167
58	chloramphenicol - PC3	0.509	2	0.896	0.02231
59	oxprenolol - MCF7	-0.639	2	-0.894	0.02251
60	pentoxifylline - PC3	0.483	2	0.893	0.0236
61	methylbenzethonium chloride - PC3	-0.58	2	-0.89	0.02394
62	withaferin A - PC3	-0.608	2	-0.888	0.02511
63	tetrandrine - MCF7	-0.629	2	-0.886	0.02602
64	CP-690334-01 - PC3	-0.281	4	-0.671	0.0261
65	fulvestrant - PC3	-0.197	12	-0.407	0.0262
66	tiletamine - MCF7	0.474	2	0.885	0.0272
67	levomepromazine - MCF7	-0.626	2	-0.882	0.02791
68	paclitaxel - MCF7	0.358	3	0.757	0.0285
69	canrenoic acid - PC3	-0.549	2	-0.88	0.02907
70	acacetin - PC3	-0.589	2	-0.877	0.03044
71	triamterene - PC3	-0.56	2	-0.876	0.03094
72	sulfadimidine - PC3	0.422	2	0.873	0.03245
73	metformin - PC3	0.567	2	0.871	0.03354
74	sulfinpyrazone - MCF7	-0.635	2	-0.869	0.03406
75	chlorcyclizine - PC3	-0.558	2	-0.869	0.03438
76	thioridazine - HL60	-0.464	4	-0.652	0.03467
77	thioguanosine - MCF7	-0.658	2	-0.868	0.03469
78	3-nitropropionic acid - PC3	0.469	2	0.868	0.03521
79	dobutamine - MCF7	0.413	2	0.868	0.03543
80	zidovudine - MCF7	0.221	2	0.864	0.0376
81	propranolol - MCF7	0.343	2	0.864	0.03772
82	CP-690334-01 - MCF7	-0.374	4	-0.645	0.03796
83	parbendazole - PC3	0.223	2	0.86	0.03964
84	dilazep - PC3	-0.54	2	-0.857	0.04088
85	fenoprofen - MCF7	0.439	3	0.723	0.04172
86	PHA-00745360 - MCF7	-0.238	4	-0.637	0.04182
87	probenecid - MCF7	0.182	2	0.856	0.04191
88	alpha-ergocryptine - MCF7	-0.458	3	-0.726	0.04232
89	procarbazine - MCF7	0.222	2	0.855	0.04243
90	sulfapyridine - MCF7	0.273	2	0.855	0.04286
91	raubasine - MCF7	0.231	2	0.854	0.04308
92	fenbendazole - PC3	0.26	2	0.854	0.04338
93	pioglitazone - PC3	-0.233	5	-0.57	0.04408

94	copper sulfate - MCF7	-0.56	2	-0.851	0.04414
95	kanamycin - MCF7	-0.547	2	-0.85	0.04481
96	amikacin - MCF7	-0.547	2	-0.85	0.04485
97	diltiazem - MCF7	0.209	2	0.846	0.0477
98	cefsulodin - MCF7	0.193	2	0.844	0.04871
99	quercetin - MCF7	-0.467	4	-0.625	0.04894
100	nitrendipine - MCF7	-0.339	3	-0.711	0.04927
101	pimozide - MCF7	-0.584	2	-0.843	0.04966
102	cyclobenzaprine - MCF7	-0.662	2	-0.842	0.04994
