# Supplementary material for: Transcriptome signature analysis repurposes trifluoperazine for the treatment of fragile X syndrome in mouse model
Source: Commun Biol. 2020 Mar 16;3:127. doi: 10.1038/s42003-020-0833-4 (PMC7075969; doi:10.1038/s42003-020-0833-4)
Supplement: Supplementary file 9 — Supplementary Data 7 [file 42003_2020_833_MOESM9_ESM.pdf]

Supplementary Data 7

Rank	Compound name and cell line	Similarity mean	N	Enrichment	P
1	fluphenazine - PC3	0.558	3	0.997	0
2	thioridazine - PC3	0.569	5	0.994	0
3	vorinostat - MCF7	0.333	7	0.92	0
4	sirolimus - PC3	0.344	8	0.871	0
5	prochlorperazine - MCF7	0.369	9	0.867	0
6	trichostatin A - PC3	0.347	55	0.862	0
7	trifluoperazine - MCF7	0.371	9	0.787	0
8	geldanamycin - MCF7	0.294	10	0.753	0
9	LY-294002 - PC3	0.315	12	0.748	0
10	trichostatin A - MCF7	0.29	92	0.729	0
11	thioridazine - MCF7	0.373	11	0.724	0
12	tanespimycin - MCF7	0.273	36	0.663	0
13	LY-294002 - MCF7	0.166	34	0.424	0
14	methylbenzethonium chloride - PC3	0.596	2	0.998	0.00002
15	astemizole - PC3	0.554	2	0.996	0.00002
16	mefloquine - PC3	0.555	2	0.996	0.00002
17	fluphenazine - MCF7	0.315	10	0.724	0.00002
18	PHA-00846566E - PC3	-0.864	2	-0.998	0.00004
19	prochlorperazine - PC3	0.525	3	0.968	0.00004
20	pyrvinium - MCF7	0.374	4	0.921	0.00004
21	fulvestrant - MCF7	0.207	21	0.511	0.00004
22	perphenazine - PC3	0.5	2	0.992	0.00006
23	tanespimycin - PC3	0.272	12	0.637	0.00006
24	phenoxybenzamine - MCF7	0.35	3	0.951	0.0001
25	thioridazine - HL60	0.327	4	0.9	0.0001
26	AR-A014418 - PC3	-0.816	2	-0.994	0.00012
27	alexidine - PC3	0.474	2	0.99	0.00012
28	promazine - PC3	0.486	2	0.989	0.00016
29	alvespimycin - MCF7	0.271	7	0.756	0.00016
30	trichostatin A - HL60	0.155	34	0.368	0.0002
31	cloperastine - MCF7	0.364	3	0.946	0.00022
32	loperamide - MCF7	0.369	3	0.943	0.00022
33	niclosamide - PC3	0.452	2	0.988	0.00026
34	lycorine - MCF7	-0.638	3	-0.94	0.00032
35	wortmannin - MCF7	0.244	10	0.608	0.00038
36	terfenadine - MCF7	0.441	2	0.985	0.0004
37	pioglitazone - PC3	-0.422	5	-0.823	0.00042
38	15-delta prostaglandin J2 - MCF7	0.301	8	0.672	0.00042
39	CAY-10397 - PC3	-0.734	2	-0.985	0.00048
40	troglitazone - MCF7	0.256	7	0.699	0.0005
41	fluspirilene - MCF7	0.423	2	0.983	0.00056
42	5707885 - PC3	0.417	2	0.982	0.00062

43	cloperastine - PC3	0.438	2	0.981	0.00062
44	sirolimus - MCF7	0.18	25	0.389	0.00066
45	trifluoperazine - HL60	0.292	4	0.853	0.00068
46	astemizole - MCF7	0.428	2	0.98	0.0007
47	rottlerin - MCF7	0.362	3	0.927	0.00072
48	beta-escin - PC3	0.422	2	0.98	0.00074
49	suloctidil - MCF7	0.427	2	0.979	0.00076
50	resveratrol - MCF7	0.288	6	0.744	0.00077
51	withaferin A - PC3	0.403	2	0.978	0.00078
52	chlorcyclizine - PC3	0.423	2	0.978	0.00082
53	Prestwick-675 - PC3	-0.835	2	-0.98	0.00085
54	perhexiline - MCF7	0.402	2	0.977	0.00087
55	clotrimazole - MCF7	0.345	3	0.924	0.00092
56	benzethonium chloride - MCF7	0.4	2	0.974	0.00107
57	azacyclonol - PC3	0.435	2	0.97	0.00159
58	methylbenzethonium chloride - MCF7	0.355	3	0.909	0.00164
59	semustine - PC3	0.381	2	0.969	0.00165
60	prenylamine - MCF7	0.392	2	0.969	0.00165
61	mefloquine - MCF7	0.381	2	0.967	0.00181
62	clozapine - PC3	0.325	3	0.903	0.00182
63	ivermectin - PC3	0.394	2	0.966	0.00191
64	niclosamide - MCF7	0.384	2	0.966	0.00205
65	Chicago Sky Blue 6B - MCF7	-0.67	2	-0.968	0.00225
66	diprophylline - PC3	-0.683	2	-0.966	0.00262
67	amitriptyline - PC3	0.422	2	0.96	0.00266
68	thiostrepton - MCF7	0.376	2	0.958	0.00304
69	pimozide - MCF7	0.368	2	0.958	0.00308
70	15-delta prostaglandin J2 - HL60	0.308	3	0.884	0.00318
71	pyrithyldione - MCF7	-0.629	2	-0.961	0.00334
72	lomustine - PC3	0.379	2	0.955	0.00358
73	monorden - MCF7	0.197	12	0.489	0.00381
74	wortmannin - PC3	0.389	2	0.95	0.00455
75	amiodarone - MCF7	0.285	3	0.865	0.00461
76	scriptaid - PC3	0.345	2	0.949	0.00481
77	mebendazole - PC3	0.357	2	0.947	0.00499
78	nortriptyline - MCF7	0.373	2	0.947	0.00505
79	nordihydroguaiaretic acid - MCF7	0.235	8	0.57	0.00536
80	iohexol - MCF7	-0.604	2	-0.95	0.00547
81	miconazole - PC3	0.358	2	0.945	0.00577
82	arecoline - MCF7	-0.579	2	-0.948	0.00588
83	disulfiram - PC3	0.35	2	0.944	0.00596
84	sulconazole - MCF7	0.346	2	0.943	0.00606
85	MG-262 - PC3	0.352	2	0.943	0.0062
86	tonzonium bromide - MCF7	0.339	2	0.942	0.0063
87	ketorolac - MCF7	-0.564	2	-0.946	0.00632
88	isoconazole - PC3	0.343	2	0.942	0.00632

89	metolazone - MCF7	-0.573	2	-0.945	0.00666
90	bepridil - MCF7	0.344	2	0.94	0.0068
91	thioguanosine - MCF7	0.355	2	0.94	0.00704
92	MS-275 - PC3	0.358	2	0.938	0.00755
93	loperamide - PC3	0.377	2	0.937	0.00765
94	vorinostat - PC3	0.37	2	0.936	0.00781
95	fendiline - MCF7	0.354	2	0.934	0.00829
96	withaferin A - MCF7	0.341	2	0.934	0.00833
97	clemizole - PC3	0.332	2	0.933	0.00849
98	clopamide - MCF7	-0.527	2	-0.935	0.00885
99	rescinnamine - MCF7	0.348	2	0.931	0.00915
100	perphenazine - MCF7	0.363	2	0.93	0.00942
101	5224221 - MCF7	0.349	2	0.929	0.00962
102	pivmecillinam - MCF7	-0.553	2	-0.93	0.01028
103	metergoline - MCF7	0.347	2	0.926	0.01046
104	protriptyline - MCF7	0.373	2	0.926	0.01058
105	ionomycin - MCF7	0.339	3	0.825	0.01074
106	quinostatin - MCF7	0.33	2	0.924	0.01127
107	isocarboxazid - MCF7	-0.506	2	-0.923	0.01205
108	isometheptene - MCF7	-0.509	2	-0.923	0.01213
109	0297417-0002B - MCF7	0.318	2	0.92	0.01247
110	clozapine - MCF7	0.136	10	0.478	0.01264
111	nadide - MCF7	-0.507	2	-0.921	0.01268
112	cyproheptadine - PC3	0.363	2	0.919	0.01278
113	trimethoprim - PC3	-0.639	2	-0.92	0.01292
114	fursultiamine - MCF7	-0.559	2	-0.92	0.01302
115	homochlorcyclizine - MCF7	0.371	2	0.919	0.01308
116	fluphenazine - HL60	0.231	4	0.716	0.01319
117	mianserin - PC3	0.339	2	0.918	0.01336
118	tetrandrine - MCF7	0.334	2	0.917	0.0136
119	disopyramide - MCF7	-0.507	2	-0.916	0.01424
120	CP-320650-01 - PC3	-0.423	4	-0.71	0.01428
121	harmol - MCF7	-0.494	2	-0.916	0.01431
122	econazole - MCF7	0.325	2	0.915	0.01435
123	proadifen - MCF7	0.337	2	0.914	0.01443
124	miconazole - MCF7	0.341	2	0.913	0.01531
125	lanatoside C - MCF7	0.257	3	0.802	0.01576
126	colecalciferol - MCF7	-0.488	2	-0.911	0.01591
127	PNU-0251126 - MCF7	-0.434	2	-0.91	0.01632
128	carbimazole - MCF7	-0.484	2	-0.908	0.01686
129	syrosingopine - MCF7	0.334	2	0.908	0.01752
130	sulfaphenazole - MCF7	-0.541	2	-0.905	0.01819
131	puromycin - MCF7	0.317	2	0.906	0.01825
132	fenbendazole - PC3	0.347	2	0.906	0.01827
133	deptropine - MCF7	0.312	2	0.904	0.01873
134	geldanamycin - PC3	0.322	2	0.903	0.01932

135	flupentixol - MCF7	0.305	2	0.903	0.01934
136	iloprost - MCF7	-0.443	2	-0.902	0.01936
137	bucladesine - MCF7	-0.218	4	-0.69	0.02003
138	parthenolide - MCF7	0.332	2	0.9	0.02058
139	flumetasone - PC3	-0.324	2	-0.897	0.02117
140	antimycin A - MCF7	0.314	2	0.898	0.02169
141	alverine - PC3	0.31	2	0.897	0.02215
142	carbamazepine - MCF7	0.229	5	0.623	0.02221
143	demeclocycline - PC3	-0.3	2	-0.893	0.02286
144	dicoumarol - PC3	-0.347	2	-0.893	0.02316
145	flunixin - PC3	-0.306	2	-0.892	0.02338
146	chlorpromazine - PC3	0.354	4	0.677	0.02379
147	oxolinic acid - PC3	-0.301	2	-0.891	0.02386
148	haloperidol - PC3	0.233	6	0.567	0.02427
149	geldanamycin - HL60	0.253	3	0.769	0.02458
150	calmidazolium - MCF7	0.346	2	0.891	0.02463
151	metitepine - MCF7	0.295	2	0.89	0.02515
152	oxetacaine - PC3	0.293	2	0.889	0.02529
153	nordihydroguaiaretic acid - PC3	0.293	2	0.887	0.0263
154	phentolamine - PC3	0.297	2	0.887	0.0264
155	sertaconazole - MCF7	0.287	2	0.887	0.02648
156	pizotifen - MCF7	0.29	2	0.886	0.02672
157	clomifene - MCF7	0.355	2	0.885	0.02714
158	dequalinium chloride - MCF7	0.297	2	0.885	0.02732
159	norcyclobenzaprine - MCF7	0.343	2	0.885	0.02734
160	thapsigargin - MCF7	0.306	2	0.882	0.02851
161	hexetidine - MCF7	0.332	2	0.881	0.02875
162	ivermectin - MCF7	0.308	2	0.88	0.02893
163	amoxapine - PC3	0.343	2	0.88	0.02899
164	felodipine - MCF7	0.247	5	0.605	0.02938
165	etofylline - PC3	-0.341	2	-0.879	0.02946
166	methotrexate - MCF7	0.248	3	0.753	0.02946
167	butoconazole - MCF7	0.285	2	0.879	0.0295
168	piperacillin - PC3	-0.398	2	-0.878	0.0298
169	oxaprozin - PC3	-0.278	2	-0.877	0.03024
170	oxetacaine - MCF7	0.291	2	0.877	0.03064
171	valproic acid - PC3	0.152	10	0.436	0.03115
172	prochlorperazine - HL60	0.246	4	0.655	0.03268
173	gossypol - MCF7	0.272	3	0.741	0.03379
174	helveticoside - PC3	0.296	2	0.869	0.03477
175	oleandomycin - PC3	-0.249	2	-0.867	0.03501
176	aminophenazone - PC3	-0.346	2	-0.867	0.03507
177	nicergoline - MCF7	0.286	2	0.868	0.03539
178	dexamethasone - PC3	-0.46	2	-0.867	0.03541
179	homatropine - PC3	-0.446	2	-0.867	0.03549
180	thiamazole - PC3	-0.275	2	-0.867	0.03569

181	clorgiline - MCF7	0.292	2	0.868	0.03591
182	naltrexone - PC3	-0.359	2	-0.866	0.03626
183	Prestwick-559 - MCF7	0.273	2	0.865	0.03694
184	dilazep - PC3	0.274	2	0.865	0.03712
185	cyanocobalamin - MCF7	-0.328	2	-0.863	0.03774
186	Prestwick-1080 - MCF7	-0.32	2	-0.862	0.03825
187	medrysone - MCF7	0.231	3	0.731	0.03834
188	Prestwick-674 - PC3	-0.257	2	-0.861	0.03849
189	quinisocaine - MCF7	0.278	2	0.861	0.03909
190	meclozine - MCF7	0.251	3	0.729	0.0393
191	6-bromoindirubin-3'-oxime - MCF7	-0.418	3	-0.732	0.04002
192	rifabutin - MCF7	0.273	2	0.857	0.04123
193	verteporfin - MCF7	0.273	2	0.856	0.04219
194	clomipramine - MCF7	0.322	2	0.855	0.04251
195	genistein - HL60	0.22	3	0.721	0.04266
196	helveticoside - MCF7	0.24	3	0.721	0.04293
197	erastin - PC3	0.273	2	0.854	0.04318
198	metixene - MCF7	0.297	2	0.852	0.04394
199	nialamide - MCF7	-0.28	2	-0.851	0.04436
200	sulfadiazine - PC3	-0.367	2	-0.851	0.04449
201	allantoin - PC3	-0.38	2	-0.85	0.04519
202	azacitidine - MCF7	0.27	2	0.85	0.04529
203	pimethixene - MCF7	0.281	2	0.847	0.04736
204	ouabain - MCF7	0.269	2	0.847	0.0475
205	beta-escin - MCF7	0.246	3	0.71	0.04761
206	sulfamethoxazole - MCF7	-0.325	2	-0.846	0.04766
207	16-phenyltetranorprostaglandin E2 - PC3	0.267	2	0.845	0.04823
208	2,6-dimethylpiperidine - PC3	-0.294	2	-0.845	0.04829
209	todralazine - PC3	-0.376	2	-0.845	0.04841
210	azathioprine - MCF7	0.212	4	0.626	0.04882
211	nicergoline - PC3	0.283	2	0.844	0.04889
212	tranylcypromine - PC3	0.276	2	0.844	0.04911
213	tolfenamic acid - PC3	0.269	2	0.844	0.04915
