# Supplementary material for: Transcriptome signature analysis repurposes trifluoperazine for the treatment of fragile X syndrome in mouse model
Source: Commun Biol. 2020 Mar 16;3:127. doi: 10.1038/s42003-020-0833-4 (PMC7075969; doi:10.1038/s42003-020-0833-4)
Supplement: Supplementary file 14 — Reporting Summary [file 42003_2020_833_MOESM14_ESM.pdf]

nature research

Corresponding author(s): Hongbing Wang

Last updated by author(s): 1-30-2020

Reporting Summary

Nature Research wishes to improve the reproducibility of the work that we publish. This form provides structure for consistency and transparency in reporting. For further information on Nature Research policies, see Authors & Referees and the Editorial Policy Checklist.

Statistics

For all statistical analyses, confirm that the following items are present in the figure legend, table legend, main text, or Methods section.

n/a	Confirmed	
☐	☒	The exact sample size (*n*) for each experimental group/condition, given as a discrete number and unit of measurement
☐	☒	A statement on whether measurements were taken from distinct samples or whether the same sample was measured repeatedly
☐	☒	The statistical test(s) used AND whether they are one- or two-sided<br>*Only common tests should be described solely by name; describe more complex techniques in the Methods section.*
☒	☐	A description of all covariates tested
☐	☒	A description of any assumptions or corrections, such as tests of normality and adjustment for multiple comparisons
☐	☒	A full description of the statistical parameters including central tendency (e.g. means) or other basic estimates (e.g. regression coefficient) AND variation (e.g. standard deviation) or associated estimates of uncertainty (e.g. confidence intervals)
☐	☒	For null hypothesis testing, the test statistic (e.g. F, t, r) with confidence intervals, effect sizes, degrees of freedom and P value noted<br>*Give P values as exact values whenever suitable.*
☒	☐	For Bayesian analysis, information on the choice of priors and Markov chain Monte Carlo settings
☒	☐	For hierarchical and complex designs, identification of the appropriate level for tests and full reporting of outcomes
☒	☐	Estimates of effect sizes (e.g. Cohen's *d*, Pearson's *r*), indicating how they were calculated

Our web collection on statistics for biologists contains articles on many of the points above.

Software and code

Policy information about availability of computer code

Data collection	*Provide a description of all commercial, open source and custom code used to collect the data in this study, specifying the version used OR state that no software was used.*
Data analysis	SPSS 11.5 for Windows was sued for data analysis. Image J (NIH, MD, USA) was used to quantify signal in Western blot.

For manuscripts utilizing custom algorithms or software that are central to the research but not yet described in published literature, software must be made available to editors/reviewers. We strongly encourage code deposition in a community repository (e.g. GitHub). See the Nature Research guidelines for submitting code & software for further information.

Data

Policy information about availability of data

All manuscripts must include a data availability statement. This statement should provide the following information, where applicable:

- Accession codes, unique identifiers, or web links for publicly available datasets
- A list of figures that have associated raw data
- A description of any restrictions on data availability

Accession number of RNAseq data is provided. Raw data for all graphs are provided. Full Western blot images are provided.

Field-specific reporting

Please select the one below that is the best fit for your research. If you are not sure, read the appropriate sections before making your selection.

☒ Life sciences ☐ Behavioural & social sciences ☐ Ecological, evolutionary & environmental sciences

Life sciences study design

All studies must disclose on these points even when the disclosure is negative.

Sample size	Sample size was estimated by power analysis (80% power and probability level of 0.05 along with the anticipated effect size) or based on previous reports.
Data exclusions	There was no data exclusion.
Replication	Data were collected using multiple batches of culture and mice from multiple litters. Data were reproducible.
Randomization	The control and treatment groups were randomly chosen.
Blinding	Experimenters were blind to the treatments and genotypes.

Reporting for specific materials, systems and methods

We require information from authors about some types of materials, experimental systems and methods used in many studies. Here, indicate whether each material, system or method listed is relevant to your study. If you are not sure if a list item applies to your research, read the appropriate section before selecting a response.

Materials & experimental systems

n/a	Involved in the study	
☐	☒	Antibodies
☒	☐	Eukaryotic cell lines
☒	☐	Palaeontology
☐	☒	Animals and other organisms
☒	☐	Human research participants
☒	☐	Clinical data

Methods

n/a	Involved in the study	
☒	☐	ChIP-seq
☒	☐	Flow cytometry
☒	☐	MRI-based neuroimaging

Antibodies

Antibodies used	Anti-S6K1 (Cat #2708, 1:1000 dilution), anti-phospho-S6K1 (at Thr389) (Cat # 9234, 1:1000 dilution), anti-phospho-Akt (at Ser473) (Cat # 4060, 1:1000 dilution), anti-Akt (Cat # 9272, 1:1000 dilution), anti-acetylated histone H3 at K9 (Cat #9649P, 1:1000 dilution), anti-acetylated histone H4 at K8 (Cat #2594P, 1:1000 dilution), anti-histone H3 (Cat #4499P, 1:1000 dilution), and anti-histone H4 (Cat #13919P, 1:1000 dilution) were from Cell Signaling. ?-actin antibody was from Sigma (Cat #A5441, 1:10,000 dilution). Anti-FMRP (mouse monoclonal antibody 2F5, 1:1000) was a generous gift from Dr. Jennifer Darnell at the Rockefeller University. Anti-puromycin antibody (Cat # EQ0001, 1:1000) was from KeraFAST.
Validation	These antibodies have been validated by the companies and published work. They were further validated by checking the signals molecular weights. Anti-FMRP antibody was validated with FMRP knockout samples.

Animals and other organisms

Policy information about studies involving animals; ARRIVE guidelines recommended for reporting animal research

Laboratory animals	The wild type and Fmr1 knockout mice are in C57BL6 background.
Wild animals	NA
Field-collected samples	NA
Ethics oversight	The Institutional Animal Care and Use Committee at Michigan State University approved all procedures.

Note that full information on the approval of the study protocol must also be provided in the manuscript.
